# Imaging the neural circuitry and chemical control of aggressive motivation

**DOI:** 10.1186/1471-2202-9-111

**Published:** 2008-11-13

**Authors:** Craig F Ferris, Tara Stolberg, Praveen Kulkarni, Murali Murugavel, Robert Blanchard, D Caroline Blanchard, Marcelo Febo, Mathew Brevard, Neal G Simon

**Affiliations:** 1 Center for Translational NeuroImaging, Northeastern University, Boston, Massachusetts, USA; 2 Dept Mechanical Engineering, Worcester Polytechnic Institute, Worcester, Massachusetts, USA; 3 Department of Psychology, University of Hawaii, Honolulu, Hawaii, USA; 4 Insight Neuroimaging Systems, Worcester, Massachusetts, USA; 5 Department of Biological Sciences, Lehigh University, Bethlehem, Pennsylvania, USA; 6 Department of Psychology, Northeastern University, Boston, Massachusetts 02115-5000, USA

## Abstract

**Background:**

With the advent of functional magnetic resonance imaging (fMRI) in awake animals it is possible to resolve patterns of neuronal activity across the entire brain with high spatial and temporal resolution. Synchronized changes in neuronal activity across multiple brain areas can be viewed as functional neuroanatomical circuits coordinating the thoughts, memories and emotions for particular behaviors. To this end, fMRI in conscious rats combined with 3D computational analysis was used to identifying the putative distributed neural circuit involved in aggressive motivation and how this circuit is affected by drugs that block aggressive behavior.

**Results:**

To trigger aggressive motivation, male rats were presented with their female cage mate plus a novel male intruder in the bore of the magnet during image acquisition. As expected, brain areas previously identified as critical in the organization and expression of aggressive behavior were activated, e.g., lateral hypothalamus, medial basal amygdala. Unexpected was the intense activation of the forebrain cortex and anterior thalamic nuclei. Oral administration of a selective vasopressin V_1a _receptor antagonist SRX251 or the selective serotonin reuptake inhibitor fluoxetine, drugs that block aggressive behavior, both caused a general suppression of the distributed neural circuit involved in aggressive motivation. However, the effect of SRX251, but not fluoxetine, was specific to aggression as brain activation in response to a novel sexually receptive female was unaffected.

**Conclusion:**

The putative neural circuit of aggressive motivation identified with fMRI includes neural substrates contributing to emotional expression (i.e. cortical and medial amygdala, BNST, lateral hypothalamus), emotional experience (i.e. hippocampus, forebrain cortex, anterior cingulate, retrosplenial cortex) and the anterior thalamic nuclei that bridge the motor and cognitive components of aggressive responding. Drugs that block vasopressin neurotransmission or enhance serotonin activity suppress activity in this putative neural circuit of aggressive motivation, particularly the anterior thalamic nuclei.

## Background

Aggression is a normal component of mammalian behavior [1]. For animals there is an adaptive advantage to defending a territory, fighting for limited resources, competing for mates, and protecting young. However, in the context of human behavior, the motivations, actions and limits of aggressive acts are not always clear. While social norms set the boundaries of appropriate aggressive behavior, inappropriate aggressive behavior in the form of interpersonal violence represents both a mental health and social problem [2]. As such, impulsivity and violence is studied in the context of antisocial behavior, co-morbid with DSM-defined illnesses, such as mania/depression, ADHD, PTSD, autism, and substance abuse [3,4]. Understanding the early risk factors and developmental trajectory of antisocial behavior has helped to devise effective psychosocial intervention strategies to reduce the incidence of impulsive aggression [5-9]. However, impulsivity and violence secondary to Axis I disorders appear more intractable and require both psychosocial intervention and pharmacotherapy. Unfortunately, the treatment of impulsive aggression in the clinical setting usually involves the prescription of combinations of drugs which by themselves are normally used to treat epilepsy, depression, anxiety and schizophrenia [4,10]. Hence there is a need to understand the neural mechanisms contributing to aggressive behavior for the development of targeted, behaviorally specific, pharmacotherapeutics.

Serenics are drugs [11] that reduce or delay the rapid onset of anger without impairing initiative, normal social relations, appetitive behaviors or the ability to defend oneself from challenges or threats. Historically, candidate drugs with serenic potential focused on serotonin 5HT_1a _and 5HT_1b _receptor agonists [12]. Treating excessively aggressive patients afflicted with mental retardation, brain injury or psychiatric illness with drugs like buspirone, which simulate 5HT1a receptors, or fluoxetine a selective serotonin reuptake inhibitor (SSRI), reduces several measures of aggressive responding [13-17].

While serotonin neurotransmission is associated with a reduction in agonistic behavior, vasopressin released as a neurochemical signal in the brain does just the opposite, i.e., it increases aggressive responding. There is a large body of literature reporting blockade of vasopressin V_1a _receptors in a variety of animals suppresses offensive aggression [18]. Consequently, drugs that target and block the vasopressin V_1a _receptor are being developed as potential therapeutics for the treatment of impulsivity and violence. Recently, a new class of non-peptidic compounds targeted to the human V_1a _receptor was developed using a monocyclic beta lactam platform [19]. One of these potential drugs, SRX251, was tested for serenic activity in the hamster resident/intruder paradigm of offensive aggression [20]. Oral administration of SRX251 caused a dose-dependent decrease in several measures of aggressive behavior without affecting motor activity, olfactory communication, and sexual motivation.

Normal aggressive behavior and aggression characterized by impulsivity and violence are envisioned to be organized and controlled by a distributed neural circuit, i.e., subsets of interconnected neurons conveying sensory and motor information to and from sites of integration [21]. By all accounts, this neural circuit is plastic, subject to modification by past experience as well as present environmental and endocrine factors that influence the probability and intensity of an agonistic encounter [22]. Our present understanding of this putative neural circuit controlling aggressive behavior is based on early studies using chemical and electrical stimulation and lesion techniques in discrete brain areas [23-25]. More recently, immunostaining for immediate early gene proteins as cellular markers of neuronal activity helped identify multiple areas across the brain presumably involved in aggressive behavior [26-32]. Unfortunately, the temporal window for these cellular markers is 50–60 min after the agonistic encounter leaving in doubt the precise onset and location of neuronal activity associated with the start of an aggressive attack. Newer imaging technologies like functional magnetic resonance imaging (fMRI) with the blood oxygen level-dependent (BOLD) technique may resolve this problem because it is possible to acquire data on changes in brain activity in seconds. As part of the ethogram of aggression, male rats in the company of their female cage mate will piloerect the fur along the midline back in the presence of a male intruder. This piloerection is unique to offensive aggression, is not seen in other behaviors, and signals an impending attack on the intruder [33]. These characteristics of piloerection combined with its occurrence very early in the aggression ethogram, overcome the limitations that are associated with motion artifacts that can be seen with fMRI [34]. For the present studies, we developed a tube shaped vivarium that fits into the bore of the magnet within centimeters of the eyes and nose of the male being imaged. This vivarium can accommodate the female cage mate and the introduction of a novel male competitor. We discovered that even though a resident male is confined to a restraining device for an imaging session, placing an intruder into the vivarium with its cage mate induces piloerection – the peripheral, autonomic sign of aggressive motivation. Because head restraint is a limitation in any awake animal fMRI study, it is not possible to image the neural circuit involved in the consummatory aspects of aggression like attacks and bites. However, with the present experimental approach we report that it is possible to identify the distributed putative neural circuit associated with the genesis of attack behavior. In addition, the technique also allows the activity of this neural circuit to be imaged in the presence or absence of different drug treatments that affect aggressive responding.

## Results

**Figure 1:**
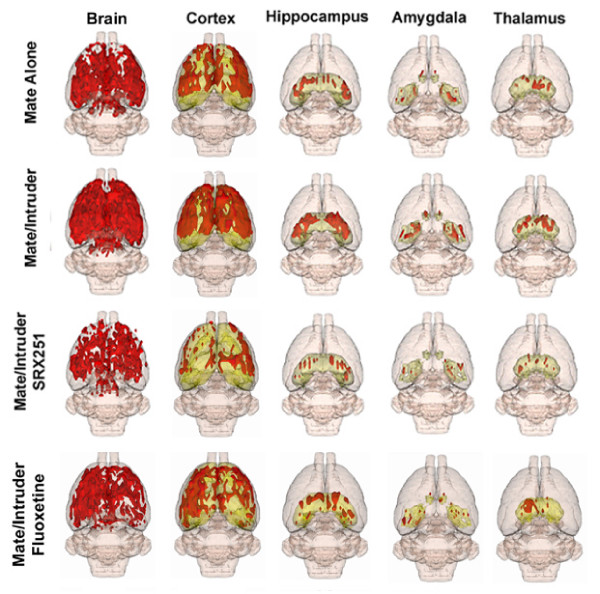
**Three dimensional representations of BOLD activation**. The pictures show translucent shells of the brain viewed from a caudal/dorsal perspective. The red depicts the localization of activated voxels interpolated into a 3D volume of activation for four experimental conditions: mate alone, mate/intruder, and pretreatment with SRX251 or fluoxetine, followed by the aggressive promoting stimulus of mate/intruder. The volumes of activation for each experimental condition are composed of 10 male residents each. Once fully registered and segmented, the statistical responses for each animal are averaged on a voxel-by-voxel bases. Those averaged voxels that are significantly different from baseline and exceed a 2.0% threshold are show in their appropriate spatial location. The volumetric data shown in the whole brain 3D models on the left column were parsed into the four major brain areas noted. The geometric volumes constituting each major area like the hippocampus, i.e., subiculum, dentate gyrus, CA1, CA2, CA3 have been melded into a single volume shown in yellow.

**Figure 2:**
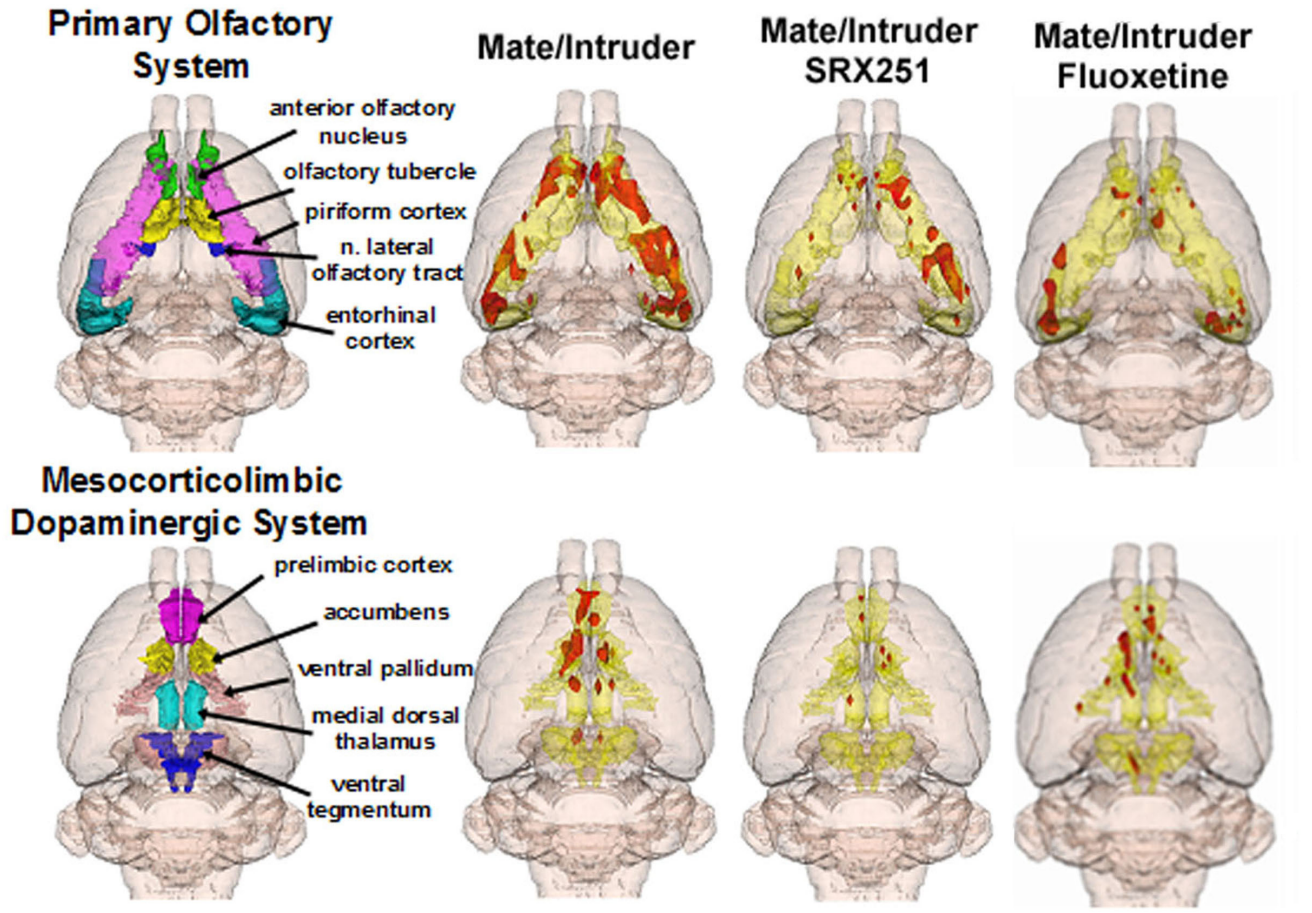
**Activation of functional neuroanatomical systems**. Shown is a reduction in activity in the primary olfactory and mesocorticolimbic dopaminergic systems in response to mate/intruder following SRX251 and fluoxetine treatment. These 3D volumes of activation are composed of 10 subjects each.

The total volume of brain activation for resident males confronted with their mate alone, mate plus intruder, mate plus intruder in the presence of V1a receptor blockade (SRX251), or fluoxetine can be viewed as 3D models (Fig 1). These 3D volumes of activation from the four experimental groups are a composite of ten subjects each and provide a visual representation comparing the difference in the number of activated voxels across experimental conditions. There is an ostensible increase in brain activity (far left column) with the presentation of the intruder as compared to the mate alone. This brain activity is reduced with SRX251 treatment in the presence of the intruder, but less so with fluoxetine treatment. This profile of activation is similar across major brain regions, e.g. cortex, amygdala, hippocampus, and thalamus (Fig 1). There appears to be a general decrease in BOLD signal in all major regions with SRX251 but less so for fluoxetine treatment as compared to the activity observed with mate alone or mate/intruder. This drug-induced pattern of brain activity in response to aggression-promoting cues also extends to functional neural circuits like the olfactory system and the reward pathway. Figure 2 shows 3-D images of the activation pattern in the primary olfactory and mesocorticolimbic dopaminergic systems for aggressive motivation alone (mate/intruder), and SRX251 or fluoxetine in the presence of mate/intruder. Activation of both neural circuits is most apparent during mate/intruder stimulated aggressive motivation. Moderate activity is still present with fluoxetine treatment but SRX251 appears to suppress all activity in both neural circuits.

**Figure 3:**
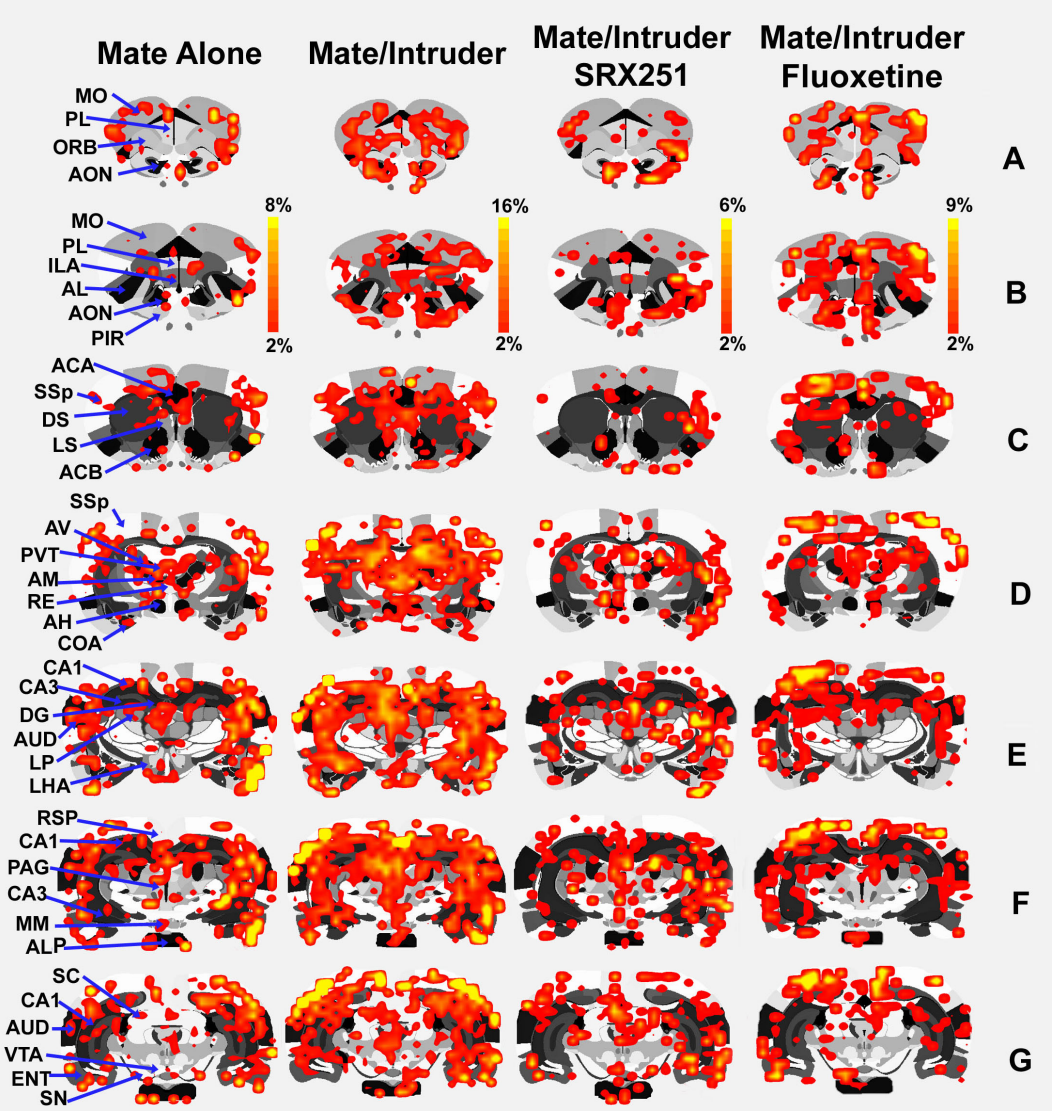
**Coronal 2D BOLD activation maps**. Shown are activation maps for each experimental condition localized to coronal sections of the segmented rat atlas. The red/yellow depicts the localization of significantly activated and interpolated voxels that exceed a 2% threshold above baseline. The color scale denotes the percent change in BOLD signal. The areas of activation for each experimental condition are composed of 10 male residents each.

**Figure 4:**
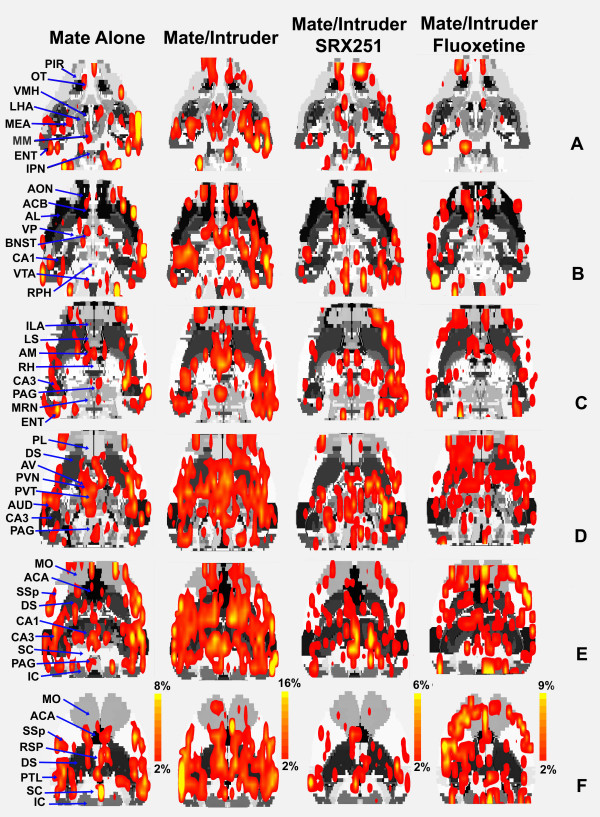
**Transverse 2D BOLD activation maps**. Shown are activation maps for each experimental condition localized to transverse sections of the segmented rat atlas. The red/yellow depicts the localization of significantly activated and interpolated voxels that exceed a 2% threshold above baseline. The color scale denotes the percent change in BOLD signal. The areas of activation for each experimental condition are composed of 10 male residents each.

BOLD activation maps, co-registered on 2-D, coronal sections for mate alone, mate plus intruder, mate/intruder with SRX251 or fluoxetine are shown in Figure 3. The same data can be viewed on 2-D transverse sections in Figure 4. These activation maps from the four experimental groups are a composite of ten subjects each, fully registered into a 3D rat MRI atlas and segmented for volumes of interest (VOI). Visual inspection of Rows A-G Fig 3 and Rows D-F Fig 4, show robust bilateral activation across the cortical mantle during aggressive motivation. Activated areas include the motor cortex (MO), primary somatosensory cortex (SSp), auditory cortex (AUD), and parietal cortex (PTL). Inspection of Row D Fig 3 and Rows C and D Fig 4 show intense activation of the anterior thalamic nuclei and dorsal midline thalamic nuclei during aggressive motivation. Activated areas include the anterior ventral (AV), medial (AM), and lateral (AL) thalamus, nucleus reunions (RH), and paraventricular thalamus (PVT). Row E Fig 3 and Row A Fig 4 show activation of the lateral hypothalamus (LHA) during aggression motivation, while Rows E-G Fig 3 and Rows C-E Fig 4 show robust activation of the hippocampus (DG, CA1, CA3).

Drug treatments caused a conspicuous change in the pattern of BOLD signal in response to mate/intruder. Residents treated with SRX251 show a reduction in signal in the somatosensory and motor cortices while fluoxetine treatment did not reduce cortical signal (B-F Fig 3; D-F Fig 4). The lateral hypothalamus shows robust activation in the presence of mate/intruder, a BOLD signal change not observed under any other experimental conditions. The ventral periaqueductal gray (F Fig 3; C Fig 4) shows a high volume of activation with mate/intruder that is less pronounced with mate alone or SRX251 treatment and is essentially absent with fluoxetine treatment. Activity in the raphe nucleus (B Fig 4) is absent in animals treated with fluoxetine.

**Figure 5:**
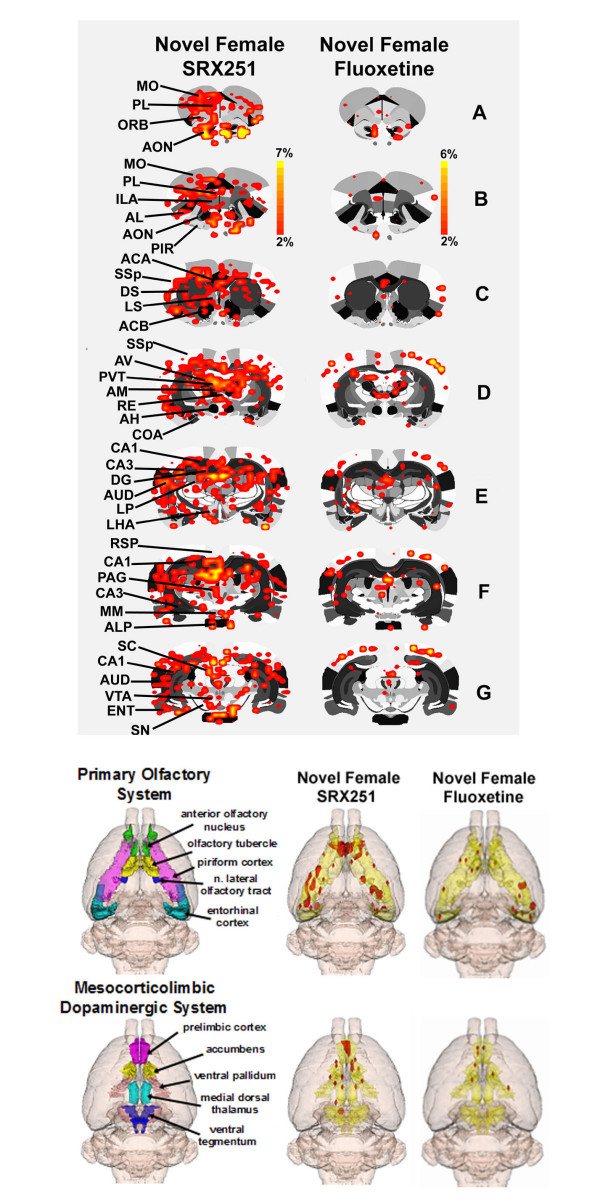
**Activation maps for sexual stimulation**. Shown above are 2D BOLD activation maps localized to coronal section of the segmented rat atlas for SRX251 and fluoxetine treatment in response to a novel receptive female. The red/yellow depicts the localization of significantly activated and interpolated voxels that exceed a 2% threshold above baseline. The color scale denotes the percent change in BOLD signal. The areas of activation for each experimental condition are composed of 10 male residents each. Below are 3D volumes showing the pattern of activation to a novel receptive female in the olfactory and reward systems following SRX251 and fluoxetine treatment. These 3D volumes of activation are composed of 10 subjects each.

The general diminution in BOLD activity with SRX251 during aggressive motivation raises questions about drug specificity and the apparent suppression of olfaction as a reason for the decrease in responsiveness. However, when SRX251 treated males are presented with a novel receptive female during image acquisition they show good activation of the primary olfactory system and reward pathway as shown in Fig 5. This activation of the primary olfactory system in the presence of SRX251 in response to a receptive female but not during aggressive motivation attests to the specificity of drug action and the stimulus-dependent nature of BOLD activation. The same is true for fluoxetine as the profile of BOLD activation is lowest toward a receptive female and highest toward an intruder. Indeed, there is a noticeable absence of brain activity toward a receptive female with fluoxetine treatment (Fig 5).

**Figure 6:**
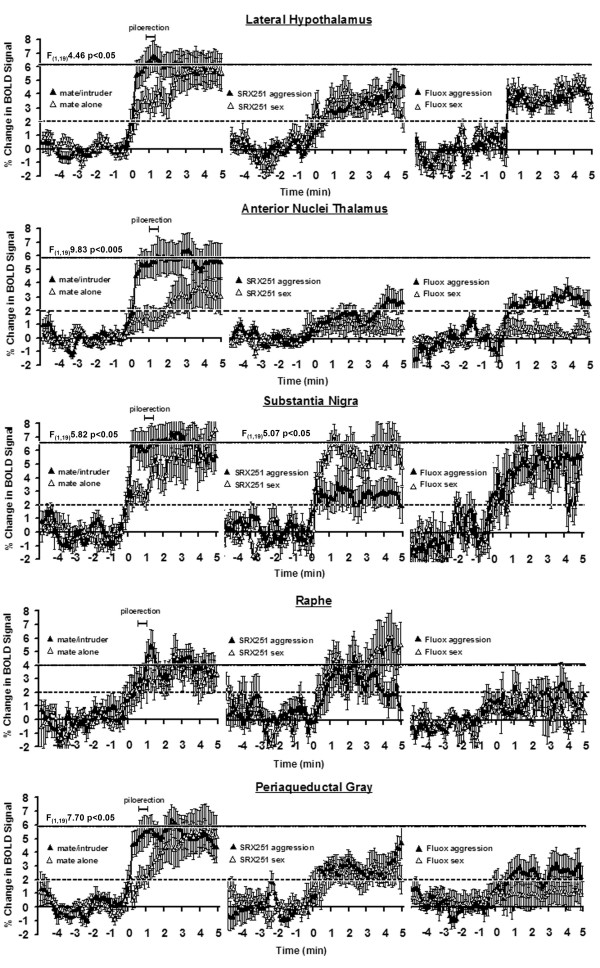
**Change in BOLD signal over time**. Shown are representative time course data from select brain areas depicting the change in BOLD signal following stimulus presentation (time 0 min). For each brain area the data in response to mate alone or mate/intruder are presented on the left column while the data from mate/intruder (aggression) or novel receptive female (sex) in the presence of SRX251 or fluoxetine are presented in the middle and right columns. The dashed horizontal line marks the threshold of 2% below which is baseline noise in awake imaging studies. The solid line at top represents the approximate BOLD signal change for aggressive motivation alone. The scale marked piloerection shows the range of time (62 ± 11) for piloerection following introduction of the mate/intruder into the vivarium. The percentage change in BOLD signal intensity at each time point (100 data acquisition over the 10 min scanning period) is the average of 10 male residents for each experimental condition. Vertical lines at each data point denote the standard error of the mean. Where show, the F values represent significant differences between experimental conditions based on a repeated measured 2-way ANOVA for the 1^st ^min post stimulation.

**Figure 7:**
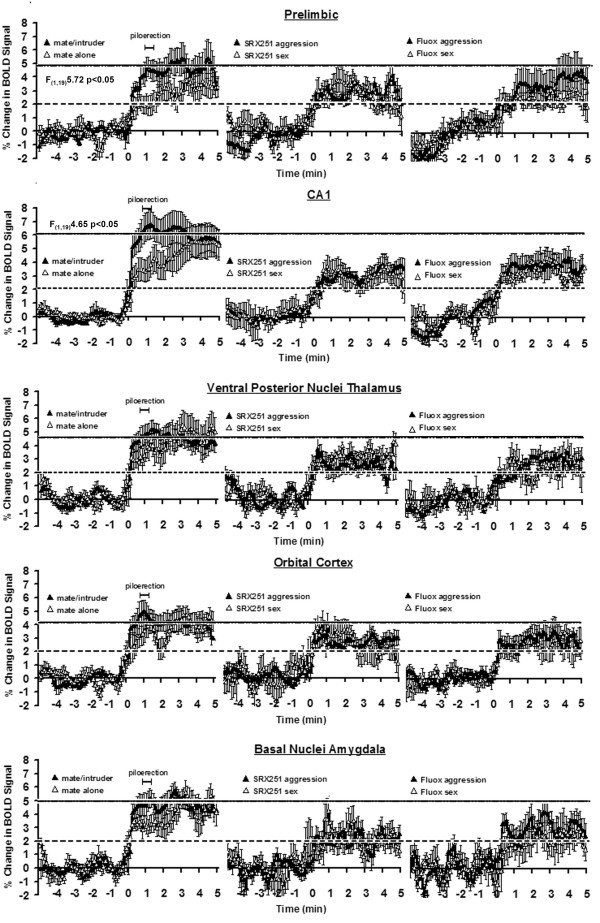
**Change in BOLD signal in key areas of interest**. See legend from figure 6.

**Figure 8:**
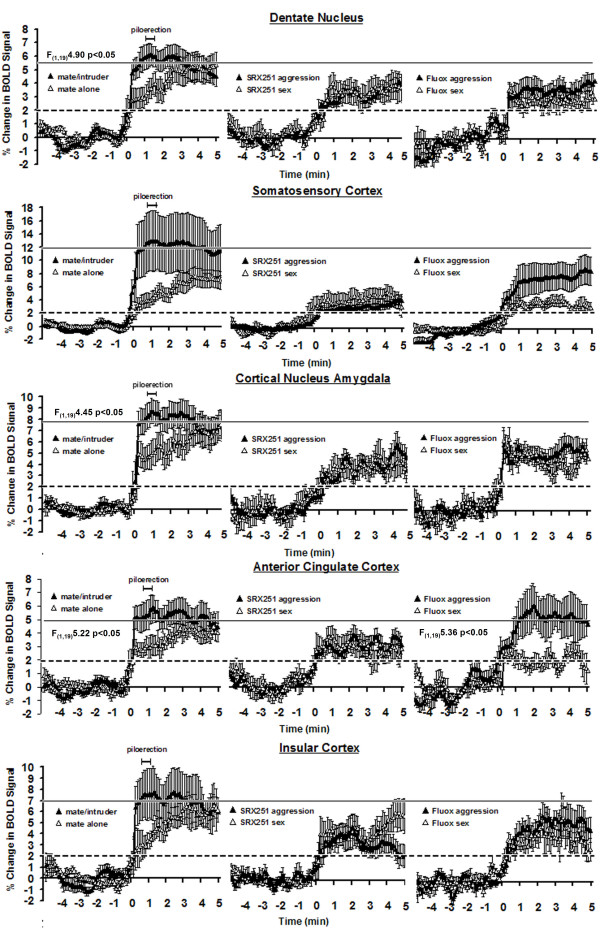
**Change in BOLD signal in limbic cortical areas**. See legend from figure 6.

The putative neural circuit of aggressive motivation was determined by comparing the volume of activation (i.e. number of voxels) between mate and mate/intruder using a Wilcoxon signed-rank test. These brain areas include the somatosensory, auditory, orbital and retrosplenial cortices, hippocampus, several amygdaloid nuclei and bed nucleus of the stria terminalis, anterior thalamic nuclei, paraventricular and lateral hypothalamus, and prelimbic cortex as shown in Table 1. These sixteen sites were identified from eighty-three brain areas. Brain areas associated with aggressive motivation were not only identified by the volume of activation but were also characterized by a rapid and robust increase in BOLD signal intensity that peaked near the time of piloerection (Table 1). These brain areas include the retrosplenial (F_(1,19) _= 5.42, p < .03) and prelimbic cortex (F_(1,19) _= 5.72, p < .03), CA1 (F_(1,19) _= 4.65, p < .04) and dentate gyrus (F_(1,19) _= 4.90, p < .04) of the hippocampus, cortical amygdala (F_(1,19) _= 4.45, p < .05), lateral posterior (F_(1,19) _= 6.66, p < .02) and anterior (F_(1,19) _= 9.83, p < .005) thalamic areas and lateral hypothalamus (F_(1,19) _= 4.46, p < .05). In contrast, brain activation in response to the presence of the mate alone showed a gradual increase in BOLD signal that reached the same magnitude seen with aggressive motivation (left columns Figs 6, 7, 8). Indeed, repeated measures, 2-way ANOVA comparing the entire five min post stimulus period for mate vs. mate/intruder showed no significant main effect between stimulus conditions, but a significant main effect for time. For example, the change in BOLD signal over time for the lateral hypothalamus (Fig 6) showed no statistical difference between stimulus conditions (F_(1,19) _= 1.05, p = 0.318) but a significant difference in the change of BOLD signal over time (F_(49,980) _= 2.19 p < .0001). The only exception to this pattern of activation in the putative neural circuit of aggressive motivation was the anterior thalamic area (Fig 6) which showed significant main effects for condition (F_(1,19) _= 5.07, p < .04) and time (F_(49,980) _= 2.19 p < .002).

**Table 1:** Activation of brain areas associated with aggressive motivation

	**Volume of Activation**		**% Change in BOLD Signal**	
**Brain Area**	**Mate Alone**	**Mate + Intruder**	**Mate Alone**	**Mate + Intruder**
retrosplenial cortex	26 (10, 61)	50 (26, 99)**	5.0 ± 0.3	10.5 ± 0.5*
orbital cortex refs [30,31,203]	9 (1, 14)	19 (4, 44)*	3.0 ± 0.2	4.1 ± 0.2
auditory cortex	20 (4, 61)	38 (23, 51)*	4.3 ± 0.5	10.1 ± 0.6
somatosensory cortex	114 (16, 266)	221 (141, 392)*	3.7 ± 0.3	11.9 ± 1.1
prelimbic cortex refs [30,31,203-205]	2 (0, 11)	8 (2, 25)*	1.7 ± 0.1	3.5 ± 0.2*
CA1 hippocampus refs [21,30]	27 (10, 67)	46 (32, 110)*	3.1 ± 0.2	5.7 ± 0.2*
dentate gyrus refs [21,30,206]	20 (3, 48)	32 (22, 80)**	2.8 ± 0.2	5.2 ± 0.2*
cortical n. amygdala refs [28,207-210]	14 (5, 29)	23 (14, 52)*	4.6 ± 0.3	7.8 ± 0.3*
basal n. amygdala ref [27]	4 (1, 11)	10 (2, 17)**	3.1 ± 0.2	4.3 ± 0.2
medial n. amygdala refs [27-29,205-207,209-216]	1 (0, 6)	3 (0, 9)*	2.7 ± 0.2	4.6 ± 0.2
bed n. stria terminalis refs [25,27,29,30,205-208,211-214,217]	2 (0, 5)	6 (2, 16)*	3.4 ± 0.3	5.1 ± 0.2
lateral post. n. thalamus	2 (0, 9)	8 (2, 11)**	1.6 ± 0.2	4.2 ± 0.2*
anterior n. thalamus	3 (0, 6)	6 (2, 12)**	1.4 ± 0.2	4.9 ± 0.2**
ventral pallidum	5 (0, 17)	11 (6, 21)*	2.2 ± 0.2	4.1 ± 0.2
lateral hypothalamus refs [25,29,32,42,44-48,178,211,212,214,217-225]	10 (3, 26)	25 (6, 54)*	3.1 ± 0.3	5.7 ± 0.2*
PVN hypothalamus refs [205-207,211,213,214,217]	1 (0, 2)	3 (1, 5)*	2.0 ± 0.1	3.8 ± 0.1

The two left columns report the median (min-max) number of voxels activated (volume of activation) in male residents when presented with their female cage mate (mate alone) or their cage mate plus a novel adult male intruder (mate + intruder). Male residents (n = 10) were tested for each condition in a counterbalanced design and the data analyzed using a Wilcoxon Signed-Rank Test. The two columns on the right report the percent change in BOLD signal (mean ± SE) for that brain area for each experimental condition. These BOLD time course data were compared by taking the first 12 data points following stimulus presentation for each condition (up to the time of piloerection) and using a repeated measures 2-way ANOVA to assess if there was a significant difference between conditions. Those areas that showed a significant increase in the number of activated voxels were screened from a data base of eighty-three brain areas and comprise the putative neural circuit of aggressive motivation. Shown in parentheses under ten of these sixteen brain areas are references from a literature review on the neuroanatomy of aggressive behavior across different species, gender and agonistic models. Note that the remaining areas not described in the animal literature are primarily from the cortex and thalamus. p < 0.05*; p < 0.01**

Table 2 reports the volume of activation and percent change in BOLD signal associated with aggressive motivation in the presence of SRX251 or fluoxetine treatment for twenty-eight of the eighty-three brain areas analyzed. These twenty-eight areas were chosen because they include the putative neural circuit of aggressive motivation (marked by bold italic) and/or show a significant difference between drug treatments. Also included in Table 2 are the ventral tegmental area and raphe because of their prominence in dopamine and serotonin neurotransmission, respectively. Both drug treatments reduce the volume of activation in many brain areas identified as part of the putative neural circuitry of aggressive motivation. However, as seen in the activation maps (Figs 1, 2, 3, 4) BOLD signal in response to aggression-promoting stimuli is ostensibly higher with fluoxetine treatment. In particular, levels of activation in many cortical areas and brain regions associated with dopaminergic neurotransmission (e.g. dorsal striatum, substantia nigra, accumbens, ventral pallidum) are significantly higher with fluoxetine treatment as compared to SRX251. There were no significant differences between treatment groups in percent change of BOLD signal over time (Table 2).

**Table 2:** Blocking BOLD signal changes following treatment with SRX251 and fluoxetine in response to aggressive promoting stimuli

	**Volume of Activation**		**% Change in BOLD Signal**	
**Brain Area**	**Mate/Intruder SRX251**	**Mate/Intruder Fluoxetine**	**Mate/Intruder SRX251**	**Mate/Intruder Fluoxetine**
***retrosplenial cortex (130)***	12 (1, 47)	24 (9, 51)**	5.2 ± 0.3	10.4 ± 0.4
***orbital cortex (45)***	3 (0, 11)	5 (0, 6)	3.3 ± 0.2	2.0 ± 0.2
***auditory cortex (98)***	11 (0, 33)	13 (4, 38)	4.0 ± 1.7	4.3 ± 0.5
***somatosensory cortex (599)***	51 (1, 132)	117 (37, 233)**	3.8 ± 0.1	5.9 ± 0.4
motor cortex (386)	24 (0, 71)	57 (20, 181)**	3.7 ± 0.2	5.6 ± 0.2
visual cortex (212)	30 (3, 74)	38 (8, 101)	5.4 ± 0.5	7.6 ± 0.9
insular cortex (128)	4 (0, 14)	10 (4, 30)*	3.1 ± 0.2	3.4 ± 0.4
***prelimbic cortex (32)***	3 (0, 4)	5 (1, 11)	2.8 ± 0.1	2.5 ± 0.2
infralimbic cortex (17)	0 (0, 3)	5 (0, 9)**	2.0 ± 0.1	1.6 ± 0.1
anterior cingulate ctx (73)	6 (0, 15)	13 (1, 32)**	3.2 ± 0.1	3.6 ± 0.3
***CA1 hippocampus (154)***	10 (0, 44)	17 (2, 63)	2.9 ± 0.3	3.3 ± 0.2
***dentate gyrus (100)***	9 (0, 27)	9 (0, 44)	2.8 ± 0.3	3.0 ± 0.3
***cortical n. amygdala (69)***	6 (0, 24)	9 (3, 20)	3.3 ± 0.2	4.6 ± 0.4
***basal n. amygdala (24)***	1 (0, 3)	3 (0, 5)	2.3 ± 0.4	2.5 ± 0.2
central n. amygdala (13)	0 (0, 1)	0 (0, 2)	1.0 ± 0.1	0.9 ± 0.1
***medial n. amygdala (14)***	1 (0, 3)	2 (0, 7)	1.5 ± 0.4	2.7 ± 0.3
***bed n. stria terminalis (15)***	0 (0, 3)	3 (0, 7)	2.5 ± 0.2	2.7 ± 0.2
***lateral post. n. thalamus (18)***	1 (0, 5)	1 (0, 6)	1.9 ± 0.2	1.5 ± 0.1
***anterior n. thalamus (12)***	0 (0, 3)	1 (0, 4	1.0 ± 0.1	2.0 ± 0.3
substantia nigra (28)	1 (0, 6)	5 (0, 6)*	2.6 ± 0.1	3.7 ± 0.3
ventral tegmentum (16)	0 (0, 4)	2 (0, 5)	0.8 ± 0.2	1.8 ± 0.2
dorsal striatum (305)	12 (0, 39)	36 (5, 147)**	2.5 ± 0.2	2.2 ± 0.2
***ventral pallidum (41)***	1 (0, 5)	3 (0, 6)*	1.3 ± 0.2	2.0 ± 0.1
***lateral hypothalamus (64)***	5 (0,15)	8 (3, 15)	2.9 ± 0.3	3.3 ± 0.3
periaquedutcal gray (42)	4 (0, 15)	3 (0, 7)	1.9 ± 0.2	3.5 ± 0.6
raphe nuclei (15)	1 (0, 6)	1 (0, 2)	2.1 ± 0.4	0.8 ± 0.1
accumbens (45)	2 (0, 5)	8 (0, 25)**	2.8 ± 0.4	3.0 ± 0.2
***PVN hypothalamus (3)***	1 (0,2)	0 (0, 2)	1.8 ± 0.1	2.0 ± 0.1

Shown are the median number of voxels (min, max) (i.e., volume of activation) and percent change in BOLD signal (mean ± SE) in different brain areas in response to aggressive motivation following SRX251 and fluoxetine treatment. These BOLD time course data were compared by taking the fifty data points following stimulus presentation for each condition and using a repeated measures 2-way ANOVA to assess if there was a significant difference between conditions. There were no significant differences in BOLD signal change over time between drug treatments. Significant differences in volume of activation between SRX251 and fluoxetine for each brain region are shown. The numbers in parenthesis shown after each brain area is the average number of voxels from ten subjects that occupy that brain volume after registration in the 3D atlas. Brain areas shown in bold italic comprise the putative neural circuit of aggressive motivation. p < 0.05*; p < 0.01**

Table 3 reports the volume of activation and percent change in BOLD signal in associated with sexual-promoting stimuli in the presence of SRX251 or fluoxetine. In contrast, to the data reported in Table 2, there is much more brain activity with SRX251 treatment than fluoxetine treatment. In particular, levels of activation in many cortical areas and hippocampus are significantly higher with SRX251 treatment as compared to fluoxetine. With the exception of the raphe nuclei, there were no significant differences between treatment groups in percent change of BOLD signal over time (Table 3). This finding was fully consistent with bench-top behavioral investigations, which showed that SRX251 blocked aggression but not sexual behavior. As noted in the Methods, a dose-response study was conducted to find a threshold dose of fluoxetine (5 mg/kg) that consistently blocked aggressive behavior. This same threshold dose of fluoxetine also blocked sexual behavior.

**Table 3:** Blocking BOLD signal changes following treatment with SRX251 and fluoxetine in response to sexual promoting stimuli.

	**Volume of Activation**		**% Change in BOLD Signal**	
**Brain Area**	**Novel Female SRX251**	**Novel Female Fluoxetine**	**Novel Female SRX251**	**Novel Female Fluoxetine**
***retrosplenial cortex (130)***	23 (3, 67) **	6 (1, 7)	6.6 ± 0.3	3.9 ± 0.2
***orbital cortex (45)***	12 (2, 45)**	4 (0, 8)	2.7 ± 0.1	2.7 ± 0.2
***auditory cortex (98)***	14 (5, 75)**	6 (1, 13)	3.3 ± 0.3	3.4 ± 0.2
***somatosensory cortex (599)***	79 (23, 292)	40 (15, 94)	3.2 ± 0.2	3.6 ± 0.2
motor cortex (386)	72 (18, 230)**	16 (6, 33)	3.1 ± 0.2	3.2 ± 0.3
visual cortex (212)	26 (3, 67)	22 (4, 63)	5.8 ± 0.5	4.2 ± 0.2
insular cortex (128)	17 (6, 62)**	6 (0, 11)	4.4 ± 0.3	3.8 ± 0.3
***prelimbic cortex (32)***	6 (1, 19)*	3 (0, 5)	2.9 ± 0.2	2.5 ± 0.2
infralimbic cortex (17)	2 (0, 13)	0 (0, 3)	2.2 ± 0.1	2.2 ± 0.1
anterior cingulate ctx (73)	15 (1, 40)**	3 (0, 10)	3.0 ± 0.2	1.9 ± 0.2
***CA1 hippocampus (154)***	20 (8, 79)**	8 (3, 15)	3.2 ± 0.2	3.4 ± 0.2
***dentate gyrus (100)***	19 (2, 47)**	4 (0, 7)	3.2 ± 0.1	2.8 ± 0.2
***cortical n. amygdala (69)***	6 (3, 26)	5 (2, 7)	4.0 ± 0.3	4.4 ± 0.3
***basal n. amygdala (24)***	1 (0, 6)	1 (0, 2)	2.1 ± 0.2	1.9 ± 0.3
central n. amygdala (13)	1 (0, 4)	0 (0, 0)	1.5 ± 0.1	1.8 ± 0.1
***medial n. amygdala (14)***	1 (0, 2)	0 (0, 1)	1.2 ± 0.3	1.4 ± 0.2
***bed n. stria terminalis (15)***	2 (0, 7)	1 (0, 14)	2.2 ± 0.2	2.0 ± 0.1
***lateral post. n. thalamus (18)***	2 (0, 8)	1 (0, 2)	2.5 ± 0.2	1.9 ± 0.2
***anterior n. thalamus (12)***	2 (0, 13)	0 (0, 7)	1.8 ± 0.2	0.6 ± 0.1
substantia nigra (28)	3 (1, 11)	1 (0, 2)	5.6 ± 0.4	4.6 ± 0.6
ventral tegmentum (16)	1 (0, 8)	1 (0, 2)	2.7 ± 0.2	2.4 ± 0.2
dorsal striatum (305)	26 (16, 158)**	6 (1, 15)	2.5 ± 0.1	2.7 ± 0.2
***ventral pallidum (41)***	2 (1, 9)	1 (0, 4)	2.1 ± 0.2	2.5 ± 0.1
***lateral hypothalamus (64)***	7 (1,24)	4 (1, 11)	3.4 ± 0.3	3.9 ± 0.3
periaquedutcal gray (42)	5 (1, 15) **	1 (0, 3)	2.9 ± 0.2	3.3 ± 0.2
raphe nuclei (15)	2 (0, 7)	0 (0, 1)	4.1 ± 0.4*	0.8 ± 0.3
accumbens (45)	5 (0, 19)	2 (0, 4)	2.2 ± 0.2	2.6 ± 0.2
***PVN hypothalamus (3)***	2 (0,6)	0 (0, 2)	1.8 ± 0.1	2.2 ± 0.1

Shown are the median number of voxels (min, max) (i.e., volume of activation) and percent change in BOLD signal (mean ± SE) in different brain areas in response to aggressive motivation following SRX251 and fluoxetine treatment. These BOLD time course data were compared by taking the fifty data points following stimulus presentation for each condition and using a repeated measures 2-way ANOVA to assess if there was a significant difference between conditions. Significant differences in volume of activation between SRX251 and fluoxetine for each brain region are shown. The numbers in parenthesis shown after each brain area is the average number of voxels from ten subjects that occupy that brain volume after registration in the 3D atlas. Brain areas shown in bold italic comprise the putative neural circuit of aggressive motivation. p < 0.05*; p < 0.01**

Time course data showing the percent change in BOLD signal intensity for select brain areas for aggressive motivation and in response to a sexually receptive female in the presence of SRX251 or fluoxetine treatment are shown in Figs 6, 7, 8. All graphs are plotted on the same scales with the exception of the somatosensory and insular cortices and cortical nucleus of the amygdala show in Fig 8. Note that each graph has a dashed line at the 2% change in BOLD signal which denotes the threshold of background, nonspecific BOLD activity routinely observed in awake rodent imaging studies. The BOLD response associated with aggressive motivation (left column) is very similar across brain areas as noted above. There is a rapid increase in activity within the first 30 sec (5 data acquisition periods) from the introduction of the intruder (time 0 min). Piloerection occurs approximately one minute after stimulus presentation and represents a period of peak activation for most areas. The time course for BOLD activation associated with aggressive motivation (black triangle) is markedly different with SRX251 and fluoxetine treatments. A solid line is shown demarking the maximal percent change in BOLD signal for aggressive motivation (mate/intruder, left column) in the drug free condition. None of the brain areas in SRX251 and fluoxetine treated conditions reached this maximum in response to either aggressive or sexual motivating stimuli. The only exceptions were the anterior cingulate cortex (Fig 8) with fluoxetine in response to aggression (mate/intruder) and the raphe (Fig 6) with SRX251 in response to sex (novel receptive female). Indeed, the temporal pattern of BOLD signal change under both drug treatments for either aggressive motivation or novel female was very similar in many brain areas (Figs 6, 7, 8). There was a rise in signal over the first 1 min exceeding the 2% threshold of background noise and stabilizing around 3–4%. The most notable exceptions to this pattern were the anterior nuclei of the thalamus, substantia nigra, raphe and periaqueductal gray (Fig 6). In the presence of SRX251, BOLD signal in the anterior thalamus failed to exceed the 2% threshold over most of the imaging period for both stimulus conditions. In the presence of fluoxetine the raphe was similarly unresponsive under both stimulus conditions as was the periaqueductal gray for receptive female. Of all the time course data in Figs 6, 7, 8, only two showed a significant difference between stimulus conditions with drug treatment, specifically the substantia nigra with SRX251 (F_(1,19) _= 5.07, p < .04) and the anterior cingulate cortex with fluoxetine (F_(1,19) _= 5.36, p < .05).

## Discussion

### Neuroanatomy of aggression

The present study describes a method for imaging aggressive motivation using piloerection as a physiological marker. This "index response" is seen in the presence of a novel male intruder together with the resident's female cohabitant and is unique to the ethogram of aggression. Of the eighty-three brain areas investigated in response to this aggression-provoking stimulus, sixteen showed a significant increase in the volume of activation over the mate alone. We propose that these sixteen areas comprise the distributed neural circuit involved in the control of aggressive motivation. Historically, several of these areas, including the lateral hypothalamus, cortical and medial amygdala, and bed nucleus of the stria terminalis, have a key role in the control of aggressive responding (see Table 1 for references). Their place in the neural circuitry of aggression was identified by techniques using site specific electrical recordings, lesions, electrochemical stimulations, and by immunostaining for immediate early genes as biomarkers of neuronal activity. In contrast, BOLD fMRI is a noninvasive technique sensitive to the oxygenation status of hemoglobin [35]. Enhanced neuronal activity is accompanied by an increase in metabolism concomitant with changes in cerebral blood flow and blood volume to the area of activation [36-39]. Site specific changes in fMRI signal correlate with the spatial location of synaptic activity and neuronal spiking frequency [40,41]. Although fMRI lacks the spatial resolution achieved in immunostaining neurons or the millisecond temporal resolution of electrophysiology it allows repeated, real-time assessments of changes in neuronal activity across multiple brain areas. In the present study, we believe that the identified changes in BOLD signal reflect the putative neural circuit controlling aggressive motivation.

The medial basolateral hypothalamus extending from the mammillary nuclei up through the lateral and anterior hypothalamus has a fundamental role in the organization and initiation of aggressive behavior in all mammalian species studied to date. Note the robust activation of this "aggression area" in Fig 3, sections D-F, in response to mate/intruder but not to any other stimulus conditions. In these studies, the lateral hypothalamus was particularly sensitive showing both a significant increase in the volume of activation and increase in BOLD signal to aggression provoking stimuli. Electrical stimulation of the lateral hypothalamus elicits attack behavior in rats [42], cats [43], opossum [44], and monkeys [45], while electrolytic lesions in this same area reduce aggressive responding [46-48]. The lateral hypothalamus has extensive efferent connections to a majority of the brain areas that constitute the putative neural circuit of aggressive motivation identified with fMRI (see Table 1). Anterograde tract tracing studies show extensive monosynaptic connections to the paraventricular nucleus of the hypothalamus (Fig 4D), ventral pallidum (Fig 4B), medial, cortical and basal nuclei of the amygdala (Fig 3D & Fig 4A), bed nucleus of the stria terminalis (Fig 4B), CA1 of the hippocampus (Fig 3E–G & Fig 4B, E), and prelimbic/infralimbic cortex (Fig 3B) [49-52]. Given the pivotal position of the lateral hypothalamus in the neural circuitry of aggression, the *a prior *hypothesis, routinely used in fMRI studies, predicted activation of this brain area.

In our findings, the cerebrum, particularly the somatosensory, auditory, orbital and retrosplenial cortices are activated with aggressive motivation (Fig 1, Fig 3A, E–G, Table 1). These cortical areas are conspicuously absent from the literature describing the neuroanatomy of aggression in animals (see references Table 1) and are devoid of direct afferent connections from the lateral hypothalamus. However, they are consonant with an extensive human neuroimaging literature indicating that aggression-inducing stimuli produce activation in frontal cortex sites [53-55]. This cortical activation is typically interpreted as indicating that frontal cortex exhibits inhibitory control over a number of strongly motivated behaviors, including both aggression and defense, a suggestion that is supported by the relationships between prefrontal cortex and subcortical structures involved in these emotional responses [56] as well as by the effects of frontal cortex lesions [57] and damage ([58] for review) on impulsivity and aggression.

In addition to involvement of inhibition-linked areas of frontal cortex, the primary motor cortex may be involved in direct activation of the behaviors involved in offensive attack behavior. Offensive attack is a complex and precisely targeted behavior pattern [33,59]. Separation of cortical and subcortical structures, or extensive cortical damage [60] downgrade this pattern and alter or abolish its targeting, suggesting that the behaviors involved in the offensive attack pattern are organized as are other complex, voluntary behaviors, through the primary motor cortex. Although the rats in this study were restrained and physically unable to attack, efforts to do so would be expected to produce much the same pattern of motor cortex activity as an actual attack. The robust activation that occurred in the somatic sensory cortex just posterior to frontal cortex may reflect somatic sensations associated with attempts to make attack movements, or, with sensations of piloerection in animals tightly confined in a tube. Collectively, these data suggest that aggressive motivation in the context of natural stimuli is dependent upon multiple cortical areas integrating perceptual and cognitive information, possibly inhibiting or potentiating the neural circuit of aggressive motivation that ultimately leads to attack behavior.

**Figure 9:**
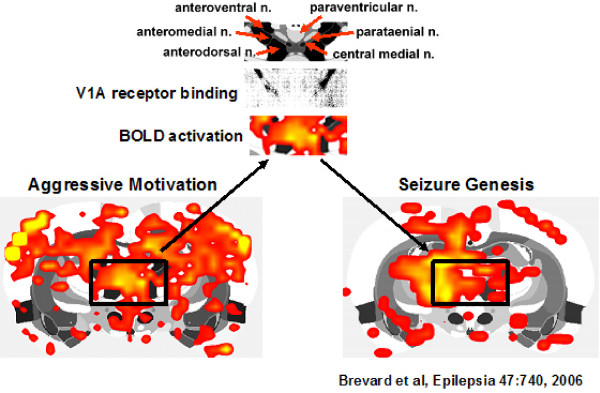
**Anterior thalamic nuclei**. Shown is a composite of figures depicting the anatomical details (top) of the dorsal midline thalamus at the level of the anterior thalamus, an autoradiograph (middle) of V1a binding density in the anterior thalamic nuclei (adapted from [100]) and BOLD activation (bottom) in the same thalamic area for aggressive motivation and seizure genesis (adapted from [67]).

The contribution of the thalamus to aggressive motivation has received little if any attention in the animal and human literature. The robust BOLD signal change in the dorsal thalamus during aggressive motivation (Fig 3D mate/intruder condition) highlights the potential importance of this area as a key trigger region in behavioral activation (see Fig 9). This area of the dorsal thalamus comprises multiple midline nuclei, e.g., paraventricular, central medial, paratenial, medial dorsal, paracentral and medial habenula bordered by the anterior thalamic nuclei, e.g. anteroventral, anteromedial and anterodorsal. When compared to mate alone, only the anterior thalamic nuclei exhibited a robust increase in BOLD signal (Fig 3D &4C, D) and volume of activation (Table 1) in response to mate/intruder. Treatment with SRX251 or fluoxetine dramatically attenuated the increase in BOLD signal change in the anterior thalamic nuclei in response to aggressive or sexual stimuli (Fig 6). The BOLD signal change did not exceed the 2% baseline threshold over most of the imaging period in the presence of SRX251 and only marginally so for fluoxetine with aggressive motivation but not receptive female.

The anterior thalamic nuclei have not been linked to aggressive motivation or aggressive behavior; instead, this brain area has been associated with memory, and interestingly enough, epilepsy. Lesion of the anterior thalamic nuclei disrupt recall of spatial [61] as well as nonspatial odorant memory [62] and leads to reduced Fos-like expression ('hypoactivity') in the hippocampal formation [63]. Lesion or high frequency stimulation of the anterior thalamus blocks or reduces pentylenetetrazol and pilocarpine-induced generalized seizures [64-66]. Functional imaging studies in awake rats (see Fig 9) show a robust increase in BOLD signal intensity in the anterior thalamic nuclei immediately prior to the onset of pentylenetetrazol-induced clonic seizure [67]. Based on temporal changes in BOLD signal intensity, the putative distributed neural circuit involved in the genesis of seizure includes the cortical nucleus of the amygdala, retrosplenial cortex, hippocampus particularly the dentate gyrus, and anterior thalamus. The anterior thalamic nuclei appear to be the gateway to the cortex regulating chemically induced paroxysmal electrical activity as evidenced by EEG coherence studies [68]. Indeed, the sensitivity of the anterior thalamic nuclei to electrochemical stimulation has been the rationale for electrical stimulation therapy in epilepsy patients with medically intractable seizures [69,70]

The thalamus holds a significant place in early neurology and psychology research as it was the cornerstone of the proposed neural circuitry of emotion first proposed by Walter Cannon [71] and popularized by James Papez [72]. The "Papez circuit" connects the hypothalamus and hippocampus to the limbic cortex, i.e. prelimbic, orbital, anterior cingulate, and retrosplenial cortices through the anterior thalamus. The anterior thalamic nuclei receive extensive afferent connections from the hippocampus [73,74] and the mammillary nuclei [75,76]. Anterior thalamic nuclei send primary projects to the anterior cingulate, retrosplenial, prefrontal, and orbital cortices and adjacent cortical areas all of which are activated with aggressive motivation [77-80]. The putative neural circuit of aggressive motivation reported in Table 1 includes many of the components comprising the "Papez circuit." The lateral hypothalamus has few if any monosynaptic connections to the anterior thalamic nuclei; instead the dorsal thalamic midline nuclei noted above, particularly the habenula, paraventricular and medial dorsal areas are heavily innervated. Interestingly, the mammillary body, a key area in the "Papez circuit" connecting the hypothalamus to the anterior thalamic nuclei, is not activated with aggressive motivation either by a measure of volume of activation or change in BOLD signal over time (data not shown). This does not preclude the mammillary bodies from contributing to the neural circuit of aggression. In fact, visual observation of the mammillary bodies in Fig 4A show more activation during mate/intruder than other experimental conditions. Since the many different nuclei that comprise the mammillary body, e.g. lateral & medial, mammillary nuclei, supramammillary nuclei and tuberomammillary nucleus were collapsed into a single volume for analysis, activation of any one specific substructure was obscured.

### Neurochemistry of aggression

There is a general consensus that vasopressin functions to facilitate aggressive behavior across multiple species [18]. Microinjections of vasopressin into the hypothalamus or amygdala and intraventricular administration in rodents leads to enhanced aggression while administration of a selective linear V_1a _antagonist, Manning compound [1-β-mercapto-β,β-cyclopentamethylene propionic acid 2-[0-(methyl) tyrosine] arginine vasopressin, blocks aggressive behavior [81-85]. In human and animal studies indices of aggressivity correlate with high concentrations of vasopressin in cerebrospinal fluid [86,87]. Intranasal vasopressin stimulates agonistic facial motor patterns in response to faces of unfamiliar men and biases male subjects to interpret neutral facial expressions as potentially aggressive [88,89].

Inappropriate aggressive behavior is closely correlated with changes in the neurobiology of the vasopressin system. Peripubertal hamsters socially subjugated by dominant male hamsters show altered vasopressin immunoreactivity in the hypothalamus as young adults and heightened aggression toward smaller conspecifics as compared to non subjugated littermates [90]. Newborn rat pups stressed by maternal separation show increased vasopressin fibers in the lateral hypothalamus as adults and heightened aggression as compared to littermate controls [91]. Treating adolescent hamsters with anabolic steroids increases the density of vasopressin immunoreactive fibers, V_1a _receptor and neuropeptide content in the hypothalamus and enhances vasopressin-mediated aggression in adulthood [85,92]. Peripubertal hamsters exposed to cocaine develop a highly aggressive phenotype as adults and enhanced released of vasopressin in the hypothalamus [93]. The development of dominant/subordinate relationships in hamsters causes a reduction in vasopressin levels in the hypothalamus in submissive partners [94] while dominant partners show higher levels of V_1a _binding in the hypothalamus [95]. Mice with distinct behavioral phenotypes of high and low aggressivity, show correspondingly high and low levels of vasopressin receptor density and fiber immunostaining in bed nucleus of the stria terminalis and lateral septum [96]. When high aggressive phenotypes are cross-fostered with low aggressive parents they show a reduction in aggression in a resident-intruder paradigm and lower levels of vasopressin in the bed nucleus as compared to their unfostered siblings [97].

Given the fundamental role of vasopressin in normal aggressive behavior and the evidence that adaptations in vasopressin neurotransmission to negative environmental events can foster inappropriate aggression, there is a strong rationale for the development of orally active and selective V_1a _receptor antagonists for clinical use in the control of impulsivity and violence. In the present studies, orally administered SRX251, a selective V_1a _antagonist with picomolar affinity for the human receptor [19], successfully blocked the aggressive motivation of resident males toward male intruders both on the bench-top and during an imaging session. The efficacy of SRX251 as an inhibitor of aggressive motivation, as assessed by fMRI, was characterized by a global suppression of BOLD signal expressed both as a reduction in the volume of activation (Fig 1, Table 2) and percent change in BOLD signal in areas that comprise the putative neural circuit of aggressive motivation (Figs 6, 7, 8, Table 2). This effect of SRX251 appears to be specific because when male residents were challenged with sexually motivating stimuli like the presentation of a novel receptive female, there was an increase in BOLD signal over several brain areas (Figs 5, 6, 7, 8, Table 3), many of which are not associated with the neural circuit of aggression. Indeed, the activation of the primary olfactory system and mesocorticolimbic system associated with aggressive motivation are dramatically reduced with SRX251 pretreatment (Fig 2), but these effects are not seen in the context of a sexually receptive female (Fig 5). One of the more compelling differences in brain activity between aggressive motivation and sexual stimuli following SRX251 treatment was the activation of the dopaminergic pathways originating in the substantia nigra and ventral tegmental area (Figs 2, 5, 7). SRX251 treatment suppressed activity in these areas in response to aggression-provoking stimuli but not to sexual stimuli, which may explain why sexual behavior is spared with V_1a _receptor antagonism. The substantia nigra showed a significant increase in BOLD signal over time (Fig 6), while the ventral tegmental area and its efferent connections showed an ostensible increase in the volume of activation (Fig 5). The nigrostrial dopaminergic pathway is important in sexual readiness while the mesolimbic dopaminergic pathway affects sexual motivation [98].

Brain levels of SRX251 peak within 2–4 hrs of oral administration and remain elevated for over 8–12 hrs [19]. In a previous study, we showed that the anti-aggressive effects of SRX251 were brain mediated and not due to peripheral blockade of V_1a _receptors [20]. Vasopressin V_1a _receptor binding is found throughout the brain of multiple species, particularly in many areas that constitute the putative neural circuit of aggressive motivation identified with fMRI [99-103]. Specifically, V_1a _binding is localized to the lateral hypothalamus, BNST, corticomedial amygdala, prelimbic cortex, forebrain cortex, PVN, ventral pallidum, and hippocampus. The anterior thalamic nuclei have a high density of vasopressin V_1a _receptors (Fig 9). Consequently, treating resident males with SRX251, a highly specific V_1a _receptor antagonist, could suppress aggressive responding by acting at all or some of these brain areas.

A deficit in serotonin (5-HT) neurotransmission has been implicated in many human psychiatric conditions. With respect to aggression, an inverse relationship exists between 5-HT function as measured by cerebrospinal fluid levels of the 5-HT metabolite hydroxyindoleacetic acid and conduct disorder in children, impulsivity, violence and suicide in adults [104,105]. The inappropriate aggression associated with this 5-HT deficiency trait is responsive to psychotherapeutics like the SSRI fluoxetine that increase the level of 5-HT in brain interstitial fluid. Treatments with SSRIs reduce inappropriate aggressive behavior in children and violence and impulsivity in adults [106,107]. Adult males with a history of conduct disorder show reduced measures of aggression and impulsivity when treated with an SSRI or the 5HT releasing agent D-fenfluramine [108,109]. Impulsive aggressive patients with personality disorder show blunted prolactin release after administration of fenfluramine [14], which suggests a hyposensitive 5-HT system. The correlation between low 5-HT neurotransmission or dysregulation and heightened impulsivity and aggression also is seen in non-human primates and other mammals (see review by [105]. Again, treatment with SSRIs can reduce many of the measures of aggressive and antisocial behavior.

In the present study, oral fluoxetine blocked aggressive responding in the homecage environment and piloerection during an imaging session. The anti-aggression effect of fluoxetine was characterized by an overall reduction of BOLD signal expressed as a decrease in both the volume of activation (Figs 1, 2, 3, 4, Table 2) and percent change in BOLD signal in areas that comprise the putative neural circuit of aggressive motivation (Figs 6, 7, 8). Indeed, the change in the volume of activation in response to aggression-promoting stimuli never exceeded that measured in mate/intruder alone (Table 2). The anterior nuclei of the thalamus showed an increase in BOLD signal change that just exceeded the 2% threshold (Fig 6). Interestingly, the anterior cingulate a brain area that has a major efferent connection from the anterior thalamus, showed a significant increase in BOLD signal equal to that seen with mate/intruder alone (Fig 8). One of the more compelling effects of fluoxetine treatment was the almost complete suppression of BOLD signal change in the raphe nuclei (Fig 6, Tables 2, 3). This effect may be due to the negative feedback on 5-HT neurons in the raphe through somatodendritic autoreceptors [110]. These imaging data suggest a fluoxetine mediated reduction in 5-HT neurotransmission coming from the raphe complex concomitant with an elevation of 5-HT levels at axonal nerve endings. There is evidence that chronic fluoxetine treatment down-regulates somatodendritic 5-HT1A autoreceptors at the level of the raphe enhancing 5-HT neurotransmission [111-113]. Indeed, this is has been hypothesized to explain why treatment with SSRIs for depression requires several weeks before any signs of drug efficacy [114]. To test this hypothesis with imaging we would predict that animals treated for several weeks with fluoxetine would show enhanced BOLD signal in the raphe complex.

Prior to imaging, a dose-response study determined that oral fluoxetine at 5 mg/kg was the threshold dose for blocking piloerection of resident males in their homecage environment in all subjects. At this same anti-aggressive dose, sexual motivation as measured by the latency to mount and thrust when presented with a sexually receptive female, was blocked. This suppression of sexual motivation presented as little or no change in BOLD signal with exposure to a sexually receptive, novel female. Why this stimulus would cause so little effect on brain activity as compared to aggression-promoting stimuli is uncertain. A reduction in olfactory processing may be one explanation as seen in the 3D models in Fig 5. There also is a reduction in activity in the dopaminergic mesolimbic system (Fig 5), which would have a profound effect on sexual motivation [98]. Interestingly, the lateral hypothalamus has one of the highest concentrations of 5-HT fibers and terminals in the brain [115]. Treatment with fluoxetine elevates 5-HT levels in the hypothalamus as measured by microdialysis and reduces aggressive responding in hamsters [116]. The lateral hypothalamus has 5-HT-sensitive afferent connections to the nucleus accumbens and ventral tegmental area that inhibit dopamine release in this mesolimbic system reducing sexual motivation in rats [117]. Consequently it could be hypothesized that fluoxetine by blocking the 5-HT reuptake transporter elevates 5-HT levels in the lateral hypothalamus and indirectly reduces dopamine levels in the accumbens causing both a reduction in aggressive and sexual motivation. It is well know that sexual dysfunction is an unwanted side effect of SSRI use in the treatment of depression [118]. Clinical studies point to a disruption in dopaminergic neurotransmission as the likely cause [119].

There are clear differences in brain activity toward aggressive- or sexual-promoting stimuli when the effects of SRX251 and fluoxetine are compared (Tables 2, 3). BOLD signal in response to aggression promoting stimuli with fluoxetine is greater compared to SRX251 but reduced in response to sexual stimuli. These opposite activation patterns to these highly salient stimuli point to different mechanisms of action and underscore the serenic profile of SRX251, i.e., a reduction in aggression while sparing other appetitive behaviors. Furthermore, imaging showed that both anti-aggressive drugs caused an apparent decrease in general arousal as measured by the reduction in volume of activation and BOLD signal. Previous studies in hamsters showed treatment with SRX251 selectively reduces offensive aggression without affecting motor, communicative, or sexual behaviors [20]. In the present investigation, male rats treated with SRX251 showed normal sexual motivation as measured by the latency to mount and thrust toward sexually receptive females.

**Figure 10:**
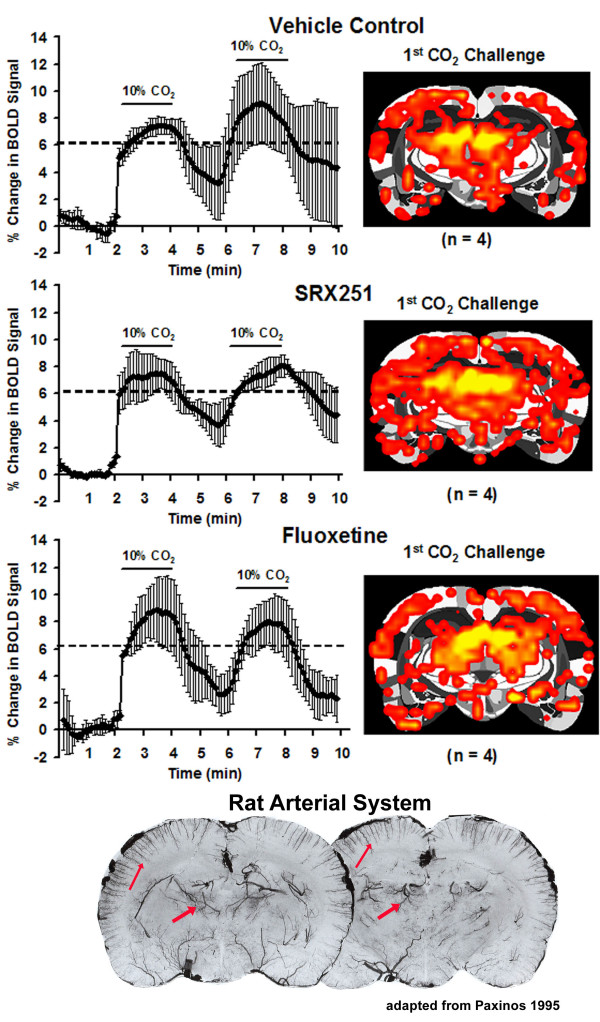
**Controlling for vascular reactivity**. Shown are time course data depicting the change in BOLD signal (mean + SD) in response to a 2 min challenge with 10% carbon dioxide (CO2). The average activation map for each experimental condition (vehicle, SRX251, fluoxetine) is shown overlayed onto the same coronal section of the segmented rat atlas. The solid horizontal line denotes the approximate maximal BOLD signal change under each experimental condition. The photomicrographs show coronal sections of the rat brain following intravascular injection of a black latex for delineating cerebral arterial blood vessels (adapted from [185]). Note the high density of vessels in the dorsal thalamus and cortex (arrows).

If the level of general behavioral activity is normal with V_1a _receptor blockade and animals show normal sexual activity, a behavior with high emotional valence, it is reasonable to ask why the BOLD signal, a hemodynamic response to metabolically active areas, was blunted? One possible explanation is the effect of SRX251 on cerebral vascular smooth muscle. Activation of V_1a _receptors on vascular smooth muscle promotes vasoconstriction and can alter blood flow. Blockade of these receptors with a highly selective antagonist might be expected to reduce vascular responsivity to endogenous vasopressin. Yet, animals treated with SRX251 and challenged with 10% CO_2 _inhalation show BOLD responses similar to untreated animals (Fig 10). This was also true for animals treated with fluoxetine (Fig 10). Another explanation for the generalized reduction in BOLD activation with SRX251 and fluoxetine is that both compounds are anxiolytics. Vasopressin V_1a _receptor antagonists and SSRIs have anxiolytic properties in several different rodent models of anxiety and stress [120-125]. Interestingly, glutamate receptor antagonists, another class of anxiolytic compounds significantly reduce the magnitude of BOLD signal in the somatosensory cortex in response to foot shock [126]. It may be that these three different classes of anxiolytics, despite their varied mechanisms of action, blunt the coupling between neuronal activity and blood flow which in this case might be reflected in a reduced BOLD response to aggressive and sexual promoting stimuli.

### Considerations in data interpretation

There are certain limitations and complications to imaging awake animals. First and foremost is the restraint of the head, without which it would be impossible to collect a clean image. Head restraint precludes the study of many behaviors that require a consummatory act, as the immobilization alone may prevent the motor response that defines the behavior. Offensive aggression as measured by the latency to bite and number of bites toward a conspecific is a case in point. However, internal states of arousal and motivation like, hunger, fear, and aggressive intent are fertile areas of investigation using fMRI and awake animals.

Key to the interpretation of these data is the association between piloerection and aggressive motivation. The value of piloerection as a signal of aggressive motivation in rats that are fixed in place for imaging and unable to show overt attack behaviors lies in consistent findings that it is the invariant precursor to offensive attack by adult male rats on other adult males [33,127]. In addition, manipulations such as castration and replacement of testosterone, that reduce and restore attack by resident males on intruders, respectively, produce similar alterations in the piloerection that precedes this attack [128]. Piloerection in an aggression context seems to be exclusively associated with offensive attack. Lesions of the amygdala that reduced defensiveness to a cat failed to alter piloerection or attack toward a male intruder [129]. Moreover, medial hypothalamic lesions that enhanced defensiveness to the experimenter, and also mouse killing by the rat subjects, produced no changes in piloerection or social aggressiveness to other male rats [130].

The stress of head restraint and restricted body movement is compounded by the noise and duration of the imaging protocols. Consequently, sensory- or drug-induced changes in MR signal in awake animals can occur against a backdrop of heightened arousal and stress – conditions that can affect data interpretation. To address these problems, protocols have been developed for acclimating animals to the environment of the MR scanner and imaging procedure leading to a reduction in stress hormones levels and measures of autonomic activity regulated by the sympathetic nervous system [131,132]. Acclimation protocols have been used to prepare awake animals for a range of behavioral, neurological and pharmacological imaging studies, including sexual arousal in monkeys [133], generalized seizures in rats and monkeys [134,135], and exposure to psychostimulants like cocaine [136-138], nicotine [139] and apomorphine [131,140]. Habituation to the scanning session is achieved by putting subjects through several simulated imaging studies. It is recognized that some stress is still likely associated with the imaging procedure. For example, piloerection in resident males typically occurs within 20 sec of introduction of the intruder male in a homecage test, while the response is delayed for up to 60 sec in the imaging environment. This delay may be due to the added complication of head restrain and stress in this experimental paradigm. Nonetheless, the piloerection occurs reliably and is highly correlated with the peak BOLD response in many brain areas.

The imaging of brain changes in response to a novel, sexually receptive female was one of the more vexing problems we encountered in these studies. In the presence of a receptive female, the male resident's teeth-chattered. This did not occur in the homecage environment, only in the imaging environment. Teeth-chattering normally occurs under stressful conditions or during psychostimulant treatments and withdrawal from drug dependence [141-143]. Teeth chattering has never been observed in any of our previous male rat imaging studies [67,132,134,138,144-149] and, interestingly is not observed in stressed animals imaged for the first time without any previous acclimation [132]. So it is unique to the imaging environment where the resident male's head is restrained and a novel sexually receptive female is just centimeters away. The teeth-chattering caused an intense level of physiological noise and despite the use of fast spin echo pulse sequences the images were distorted and the data unusable. Interestingly, resident males treated with oral SRX251 or fluoxetine did not teeth chatter, an effect we attribute to the anxiolytic activity of these drugs. Consequently, it would be incorrect to assume the neural circuitry activated in the presence of SRX251 in response to a novel receptive female is a reflection of the neural circuitry of sexual motivation alone. Comparing neural circuitry of aggressive motivation with sexual motivation is, in itself, an extremely important study, the data of which would be of interest to many in the field of psychiatry and behavioral neuroscience. However, the objective of the present study was to compare the pattern of brain activation of two drugs know to suppress offensive aggression in the resident/intruder paradigm. The sexual behavior was a control for drug specificity in the context of another highly emotional stimulus.

Positive BOLD signal changes like those reported are a function of increased cerebral blood flow (CBF), blood volume and oxygenated hemoglobin. Consequently, perturbations in mean arterial blood pressure that affect cerebral blood flow (CBF) could indirectly affect BOLD signal independent of neuronal activity. Indeed, data on BOLD imaging obtained from anesthetized rats show a clear correlation between increased mean arterial blood pressure, CBF and positive BOLD signal independent of neuronal activity (see recent papers [150-152]. This break from cerebral autoregulation in anesthetized rats where CBF becomes pressure dependent is a critical confound in pharmacological MRI ([153]. However, in a previous study from our lab [132] we reported the effect of intense autonomic arousal (increase in heart rate, body temp, and respiration) on CBF in conscious male rats. Without any acclimation, rats were restrained and imaged for regional CBF using arterial spin labeling. These same animals were then acclimated to the restraint and imaging procedure over the next several days and again imaged for regional CBF. There were no significant difference in CBF between the stress and non-stressed conditions for any brain region. These results attest to the effectiveness of cerebral autoregulation in conscious rats under extreme physiological conditions and corroborate an earlier study that showed no effect of immobilization stress on regional CBF in conscious rats [154]. It is only under conditions of severe drug-induced transient hypertension (mean arterial blood pressure exceeding 155–170 mmHg) in conscious rats were cerebral autoregulation fails and CBF becomes pressure dependent [155-158].

The use of a 3D segmented atlas with the co-registration of multiple subjects into the same volume of interest allows for region-of-interest based analyses giving measures of the volume of activation, i.e. voxel numbers, and the average percent change in BOLD signal for those activated voxels. This 3D perspective of brain function shows that stimulus-induced activation in awake animals includes an increase in BOLD signal and a recruitment of more brain volume in a region-of-interest. This data analysis reporting both a change in BOLD signal intensity and volume of activation has appeared in two of our earlier studies [137,159] and provides a unique perspective on neural coding using fMRI. Functional MRI and electrophysiology in anesthetized animals produces data that favor a labeled-line or feed forward interpretation of transmitted information. Following sensory stimulation, the relay of information to the cerebrum is reflected in a clean topographical representation at each synaptic relay of well-defined sensory receptive fields. For example, in anesthetized rats BOLD signal change in response to electrical stimulation of the skin of one foot is restricted to the contra-lateral somatosensory cortex with little or no activation outside the predicted receptive field [146,148]. However, under awake conditions, the same electrical stimulus to foot activates a greater area of the contralateral somatosensory cortex; moreover, the ipsilateral somatosensory cortex is activated [146]. The expansion of BOLD signal across a larger area of the contralateral somatosensory cortex could be explained by better neurovascular coupling as it is well know that anesthesia blunts the cortical hemodynamic response to sensory stimulation [146,148,160-163] However, the appearance of BOLD signal on the ipsilateral somatosensory cortex requires an integrated response from a distributed neural circuit.

Similarly, in electrophysiology studies, it was shown that anesthesia restricts and limits the boundaries of receptive fields [164,165]. However, simultaneous electrophysiological recordings made with multiunit electrodes from different brain areas in awake animals show a dynamic spatiotemporal quality to sensory stimulation that stretch beyond the conventional boundaries of receptive fields. The fixed topographical representation of sensory information as it ascends through the brain stem and thalamus to the cortex is replaced by distributed, integrated neural circuits that are highly flexible and can reorganize and adjust to the flow of sensory information [166-168] Levels of learning, attention or emotional arousal add another dimension to the sensory processing [169]. For example, the auditory cortex has a tonotopic or frequency map for different sounds. Tonal frequencies activate highly specific topographical areas of the auditory cortex. When these tones are associated with stimuli having emotional valence they spread over a larger cortical area not defined by the labeled line theory of neuronal coding [170,171]. Consequently, the area of activation in electrophysiology studies, or in our case, volume of activation in fMRI studies, is a critical measure in sensory processing. BOLD imaging using the volume of activation as a surrogate measure of neuronal activity is in agreement with population coding and the spread of signal across traditional boundaries as assessed with electrophysiology. Recently, it was reported that discrete micro-stimulation of the visual cortex of monkeys results in a horizontal spread of BOLD signal change that exceeds the passive spread of electrical current, despite the use of anesthesia [172]. This horizontal spread of BOLD signal in the cortex likely represents one mechanism contributing to increase in the volume of activation in fMRI studies.

## Conclusion

### The "Papez Circuit" Revisited

Early work by Paul Bard [173] showed that emotional expression or the motor components of aggression in dogs persisted following ablation of the cortex and the anterior thalamus. From experimental and clinical studies, the neuropathologist James Papez proposed a neural circuit of emotional experience or subjective feelings that included the hippocampus, anterior thalamic nuclei, mammillary body and cortex of the gyrus cinguli (e.g. anterior cingulate, retrosplenial cortex). Afferent connections from the mammillary bodies to the anterior thalamus represented the integration of emotional expression in the hypothalamus and emotional feelings in the cortex. Information from the anterior thalamic nuclei is conveyed to the gyrus cinguli where it is passed onto the forebrain cortex (e.g. prelimbic and orbital cortices) and spread laterally over the somatosensory, parietal and auditory cortices. The posterior cingulum (i.e. retrosplenial cortex) has an extensive afferent connection to the hippocampus through the angular bundle. From the hippocampus, information is conveyed to the hypothalamus and mammillary bodies through the fornix, completing the circuit. At the time this circuit was proposed the role of the hippocampus in cognitive function was uncertain. Moreover, while olfaction and the involvement of the amygdala with its connections to the stria terminalis and the lateral hypothalamus were mentioned by Papez, they were not integrated into the neural circuitry of emotion because at the time they had no ascribed function. The putative neural circuit of aggressive motivation described in this study using fMRI has the key components of the "Papez circuit" together with the olfactory circuitry and lateral hypothalamus that were missing from his original thesis. The distributed neural circuit of aggressive motivation described herein includes neural substrates contributing to emotional expression (i.e. cortical and medial amygdala, BNST, lateral hypothalamus), emotional experience (i.e. hippocampus, forebrain cortex, anterior cingulate, retrosplenial cortex) and the anterior thalamic nuclei that bridge the motor and cognitive components of aggressive responding.

### Vasopressin/Serotonin and the Control of Aggression

Enhanced serotonin neurotransmission is associated with a reduction in aggressive responding via interaction with 5-HT_1a _and 5-HT_1b _receptors. In the present study, oral fluoxetine, known to cause accumulation of 5-HT in hypothalamic intersitium, suppressed aggression and diminished BOLD activation across the putative neural circuit of aggressive motivation. Conversely, vasopressin neurotransmission promotes aggression by interacting with V1a receptors. Oral SRX251 a V1a receptors antagonist, suppressed aggression and produced a general reduction in BOLD activation in the neural circuitry of aggression similar to that seen with fluoxetine. The observation that fluoxetine and SRX251 are similar in their fMRI profile during suppression of aggressive motivation is not unexpected. There is evidence that the stimulation of aggression by vasopressin is regulated by serotonin. The hypothalamus, the primary site of vasopressinergic facilitation of aggression, has a high density of 5-HT_1a _and 5-HT_1b _binding sites and receives a dense innervation of 5-HT fibers and terminals [29,174-176]. Vasopressin neurons in the hypothalamus implicated in the control of aggression appear to be preferentially innervated by 5-HT [177]. Fluoxetine blocks aggression facilitated by the microinjection of vasopressin in the hypothalamus [116,174,178]. Fluoxetine elevates 5-HT and reduces vasopressin levels in hypothalamic tissue in hamsters [116] and rats [179]. Serotonin can also block the activity of vasopressin following its release in the hypothalamus as evidenced by the dose-dependent diminution of aggression with injections combining vasopressin and 5-HT_1a _receptor agonist. Enhanced aggression caused by activation of V1a receptors in the hypothalamus is suppressed by the simultaneous activation of 5-HT_1a _receptors in the same site [174]. Personality disordered subjects with a history of fighting and assault show a negative correlation for prolactin release in response to D-fenfluramine challenge, indication of a hyposensitive 5-HT system. These same subjects show a positive correlation between CSF levels of vasopressin and aggression [87]. Thus, in humans a hyposensitive 5-HT system may result in enhanced CNS levels of vasopressin and the facilitation of aggressive behavior.

While fluoxetine and SRX251 have similar effects on the putative neural circuitry of aggressive motivation, a markedly different fMRI signature was observed with each compound when treated males were challenged with sexual motivating stimuli. With V1a receptor blockade there was activation of the substantia nigra, ventral tegmental area and their afferent projects to the forebrain limbic cortex as well as the dorsal and ventral striatum. Measures of sexual activity in home environment were unaffected by SRX251 treatment. Treatment with fluoxetine, on the other hand, resulted in a diminished activation profile to sexual motivating stimuli and inhibition of sexual behavior in the home environment. These opposite effects point to a difference in drug specificity and underscore the serenic properties of SRX251, specifically its ability to block aggression without affecting other appetitive behaviors.

## Methods

### Animals

Adult male and female Long-Evans rats were purchased from Harlan (Indianapolis, IN, USA). Animals were housed as male/female pairs and maintained on a 12:12 hour, light: dark cycle (lights on at 7:00 hr) and provided food and water *ad libitum*. Prior to housing, all females had their oviducts ligated to prevent pregnancy. Tubal ligation was performed through a single midline incision along the abdomen while the animals were under 5% isoflurane anesthesia. The incised skin and muscle were sutured and the animals allowed several days to recover prior to any pairing. Animals were acquired and cared for in accordance with the guidelines published in the Guide for the Care and Use of Laboratory Animals (National Institutes of Health Publications No. 85–23, Revised 1985) and adhere to the National Institutes of Health and the American Association for Laboratory Animal Science guidelines. The protocols used in this study were in compliance with the regulations of the Institutional Animal Care and Use Committee at the University Massachusetts Medical School.

### Behavioral testing

**Figure 11:**
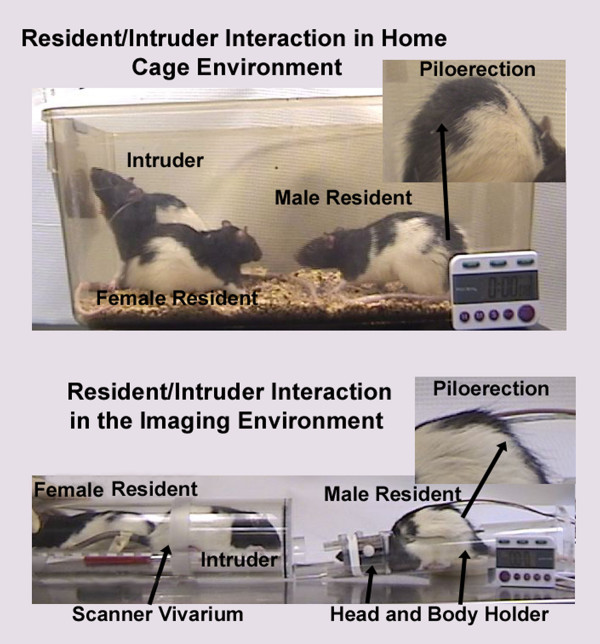
**Piloerection in the homecage and imaging environment**. The top photograph shows a male and female resident in their homecage moments after the introduction of a novel adult male intruder. The insert shows a photograph of piloerection along the midline back of the male resident. The bottom photograph shows a male resident with his head secured in the animal restrainer used for imaging. The animal's body is unrestrained and the body holder is open at the top to allow visualization of the animal's back. A vivarium designed to fit into the bore of the scanner immediately in front of the male resident is shown housing the resident's female partner and a novel adult male intruder. This presentation of stimulus animals in the vivarium elicits piloerection in the restrained male resident, an autonomic response that can be observed in the bore of the scanner during data acquisition.

The minimum duration of cohabitation between male/female pairs before testing was 2–3 weeks. On the day of an imaging session, male residents were tested for aggressive motivation by placing a novel, adult male intruder into their homecage for 5 min. It should be noted, this was the resident's only homecage encounter with a male intruder. The resident was timed for the onset of piloerection of the fur along the lower midline back (Fig 11). The average time (mean ± SD) to piloerection in the homecage environment was 18 ± 6 sec (n = 20). The "intruders" in these studies were taken from other male/female pairs. Following this homecage test, resident males were secured in the rodent restrainer used for imaging as described below. The rodent restrainer was positioned in the magnet facing an empty vivarium. Once positioned in the scanner, the resident was exposed to his female cage mate alone or his mate plus a novel intruder. The intruder was not the same animal used in the homecage test. These presentations were counterbalanced resulting in each resident being imaged twice for a total of 20 separate imaging sessions for the 10 animals. The time between the two imaging sessions was 4–5 days. During the imaging session it was possible to observe the back of the restrained resident (Fig 11) and time the onset of piloerection. The average time (mean ± SD) to piloerection in the magnet was 62 ± 11 sec. It should be noted that the vivarium was positioned in the magnet at the start of the study prior to onset of imaging.

Unique to these studies was the presentation of the stimulus animals in the magnet. To accomplish this it was necessary to design a vivarium that could be positioned in the bore of the scanner within centimeters of the male resident being imaged. The vivarium is shown in Fig 11 and consists of a Plexiglas tube 14 cm in diameter and 42 cm in length. The removable end caps of the vivarium are covered with a large mesh (0.5 × 0.5 in) copper screen allowing the occupants of the vivarium to be seen, smelled and heard by the animal being imaged. The vivarium is built with a perforated floor. Prior to an imaging session, the area beneath the floor was filled with bedding from the homecage of the male resident being imaged.

In these studies, we did not collect physiological data during an imaging session. Instead, we ran a pilot looking at changes in respiration and heart rate for resident males exposed to their female mate alone (n = 3) or their mate plus the novel male intruder (n = 3) on the lab bench under the conditions shown in Fig 11. Within the first min of mate presentation, the resident males showed a significant increase in heart rate (mean ± SD) from baseline of 402 ± 11 to 428 ± 6 (p < 0.01) and respiration (mean ± SD) from baseline of 68 ± 5 to 90 ± 13 (p < 0.01). Similarly, within the first min of mate/intruder presentation, the resident males showed a significant increase in heart rate from a baseline of 384 ± 10 to 425 ± 30 (p < 0.05) and respiration from baseline of 67 ± 8 to 81 ± 9 (p < 0.05). There were no significant differences in these measures of autonomic arousal between stimulus conditions.

### Drug treatment

In a second group of ten male/female pairs, resident males were given an oral dose of SRX251 (Azevan Pharmaceuticals, Bethlehem PA). SRX251 is a highly selective, orally active vasopressin V_1a _receptor antagonist that can cross the blood brain barrier [20]. In pilot studies, male resident rats were tested for inhibition of piloerection in response to escalating doses of SRX251 (1, 2.5, 5 mg/kg). Over a five min observation period, only the dose of 5 mg/kg blocked piloerection in all animals tested. Consequently, on the day of imaging, male residents were given an oral dose of 5 mg/kg SRX251. Approximately 90–120 min later male residents were imaged as described above. During the imaging session they were presented with a novel male intruder in the presence of their female cage mate.

In a third group of ten male/female pairs, resident male rats were given an oral dose of fluoxetine. In pilot studies, male resident rats were tested for inhibition of piloerection in response to escalating oral doses of fluoxetine (1, 2.5, 5 mg/kg). Over a five min observation period only the dose of 5 mg/kg blocked piloerection in all animals tested. Animals treated with this threshold dose of fluoxetine were imaged 90–120 min later as described above.

### Drug treatment and behavioral specificity

To test if the serenic activity of SRX251 and fluoxetine were specific to aggression and not generalized to all highly emotional stimuli associated with autonomic arousal, drug treated male residents were tested for sexual motivation in the presence of a novel female. Novel females were ovariectomized using the same surgical approach described for tubal ligation. Following recovery, animals were treated with 50 μg/kg estradiol IP for 2 consecutive days. On the third day they were given an IP injection of 500 μg/kg of progesterone and subsequently test 3–4 hrs later. This regimen of gonadal hormone treatment induces estrus, sexual receptivity and lordosis in response to tactile stimulation. In a homecage test, the resident female was removed and replaced with a novel receptive female. Resident males were tested for latency to mount the female 90–120 min following oral treatment with 5 mg/kg SRX251 or fluoxetine. The latency to mount and thrust the novel female (mean ± SD) was 92.6 ± 11.4 and 102.4 ± 11.6, (p < 0.08) for vehicle and SRX251, respectively. Animals treated with oral fluoxetine did not mount the receptive female in the five min test period. Immediately after this homecage test, male residents were imaged as described above. During the imaging session (n = 10 SRX251; n = 10 fluoxetine) they were presented with a novel sexually receptive female. It should be noted; under the present experimental conditions it was not possible to obtain clean images of brain activity in response to a receptive female without SRX251 or fluoxetine pretreatment. Interestingly, the cause of this problem was related to teeth-chattering. Teeth chattering normally occurs under stressful conditions or during psychostimulant treatments and withdrawal from drug dependent conditions [141-143]. Teeth-chattering did not occur in the homecage environment, only in the imaging environment. The teeth-chattering caused an intense level of physiological noise and despite the use of fast spin echo pulse sequences the images were distorted and the data unusable. However, in the presence of SRX251 or fluoxetine, teeth-chattering was not observed.

### Imaging awake animals

Key to imaging awake animals is controlling for motion artifact. Any minor head movement distorts the image and may also create a change in signal intensity that can be mistaken for stimulus-associated changes in brain activity [180]. In addition to head movement, motion outside the field of view caused by respiration, swallowing and muscle contractions in the face and neck are other major sources of motion artifact [181,182]. To minimize motion artifacts, studies were performed with a multi-concentric dual-coil, small animal restrainer develop for imaging awake rodents (Insight Neuroimaging Systems, LLC, Worcester MA). In brief, just prior to the imaging session, animals were anesthetized with 2–3% isoflurane. A topical anesthetic of 10% lidocaine gel was applied to the skin and soft tissue around the ear canals and over the bridge of the nose. A plastic semicircular headpiece with blunted ear supports that fit into the ear canals was positioned over the ears. The head was placed into a cylindrical head holder with the animal's canines secured over a bite bar and ears positioned inside the head holder with adjustable screws fitted into lateral sleeves. An adjustable, receive only, surface coil built into the head holder was pressed firmly on the head and locked into place. The body of the animal was placed into a body restrainer. The body restrainer "floats" down the center of the chassis connecting at the front and rear end-plates and buffered by rubber gaskets. The head piece locks into a mounting post on the front of the chassis. This design isolates all of the body movements from the head restrainer and minimizes motion artifact. Once the animal was positioned in the body holder, a transmit only, volume coil was slid over the head restrainer and locked into position.

### Acclimating animals to the imaging protocol

Animals were anesthetized with isoflurane as described above for securing the animal into the restrainer. When fully conscious, the restraining unit was placed into a black opaque tube "mock scanner" with a tape-recording of an MRI pulse sequence. This acclimation protocol lasted for 60 min in order to simulate the bore of the magnet and an imaging protocol. This procedure was repeated every other day for four days. With this procedure, rats show a significant decline in respiration, heart rate, motor movements and plasma CORT when comparing the first to the last acclimation periods [132]. The reduction in autonomic and somatic measures of arousal and stress improve the signal resolution and quality of the MR images.

### Imaging protocol

Experiments were conducted in a Bruker Biospec 4.7-T/40-cm horizontal magnet (Oxford Instrument, Oxford, U.K.) equipped with a Biospec Bruker console (Bruker, Billerica, MA U.S.A) and a 20-G/cm magnetic field gradient insert (ID = 12 cm) capable of a 120-μs rise time (Bruker). Radiofrequency signals were sent and received with the dual coil electronics built into the animal restrainer [147]. The volume coil for transmitting RF signal features an 8-element microstrip line configuration in conjunction with an outer copper shield. The arch-shaped geometry of the receiving surface coil provides excellent coverage and high signal-to-noise. To prevent mutual coil interference, the volume and surface coils were actively tuned and detuned.

Functional images were acquired using a multi-slice fast spin echo sequence. A single data acquisition included twelve, 1.2 mm slices collected in 6 sec (FOV 3.0 cm; data matrix 64 × 64; TR 1.43 sec, Eff TE 53.3 msec, TE 7 msec; RARE factor 16, NEX 1). This sequence was repeated 100 times in a 10 min imaging session consisting of 5 min of baseline data followed by 5 min of stimulation data. At the beginning of each imaging session a high resolution anatomical data set was collected using a RARE pulse sequence (12 slice; 1.2 mm; FOV 3.0 cm; 256 × 256; TR 2.1 sec; TE 12.4 msec; NEX 6; 7 min acquisition time).

### Controlling for electromagnetic interference in the vivarium model

**Figure 12:**
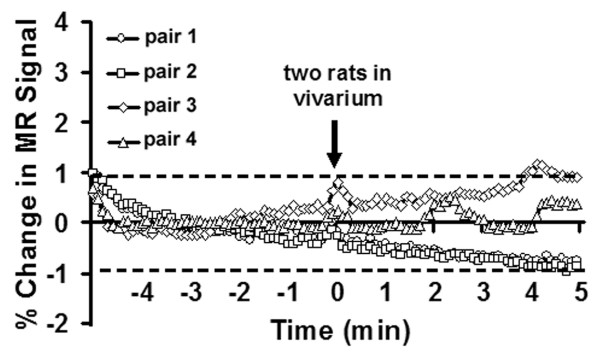
**Controlling for electromagnetic interference**. Shown are data from four experiments using a phantom to assess the change in MR signal over a 10 min imaging session when two adult male rats are added to the vivarium (arrow).

The vivarium model used in these studies is novel to the field of animal imaging. Allowing unrestrained animals to walk around a confined area in the bore of the magnet during image acquisitions raises questions about motion artifact arising from electromagnetic interference. To control for this issue a phantom positioned in the rat restrainer was imaged for a duration of 10 min consisting of 5 min of baseline data followed by 5 min following the introduction of two adult male rats into the vivarium (n = 4). The imaging protocol was identical to the fMRI protocol described above. The change in MR signal following the introduction of freely mobile rats into the bore of the magnet did not differ by more than 1% for any of the four control studies (Fig 12).

### Controlling for drift

**Figure 13:**
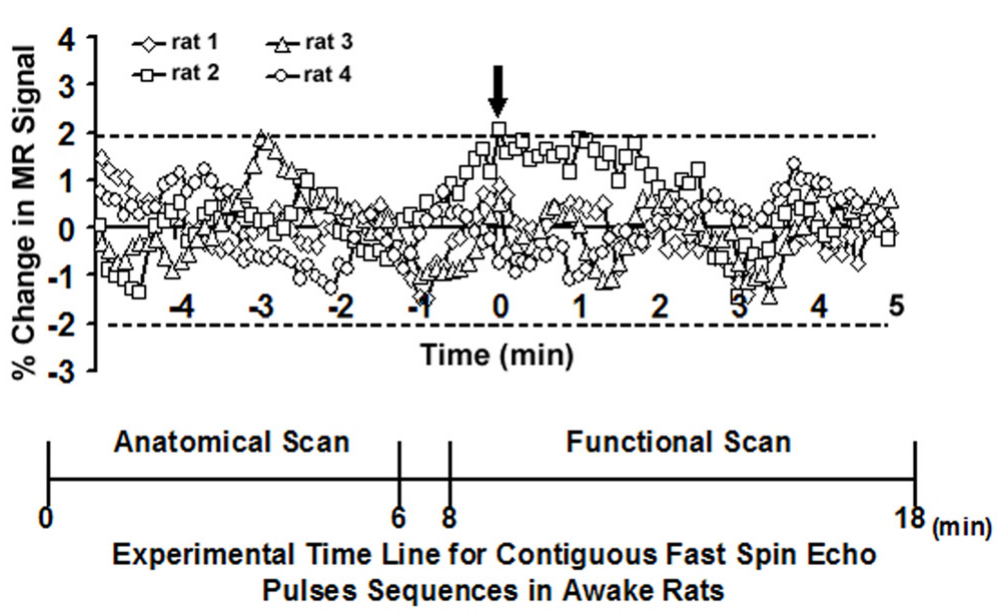
**Controlling for drift**. Shown are data from four experiments imaging a fully conscious rat without presentation of a stimulus. The arrows denote the time when a stimulus would have been introduced in a typical imaging session. The schematic shown below outlines the time course of the total scanning session including the initial high resolution anatomical data set. These data would control for any drifts associated with the instrumentation or change in the animal's physiology.

Low frequency drift is a common problem in time series fMRI studies and contributes to signal variability. Instability in temperature regulation with high performance gradients is one source of the problem; however, physiological noise and head motion are also considered to be contributing factors to drift [183]. The occurrence of false positive voxels is a concern particularly in the simple off-on activation paradigm used in these studies comparing the average baseline signal for a given voxel to its average post-stimulation signal. To control for this issue, awake rats (n = 4) were exposed to a 18 min long imaging protocol mimicking that described above for the fMRI studies. A 6 min long high resolution anatomical scan was followed two min later by a 10 min long fMRI time series. During the fMRI time series there was no stimulus presentation. The change in MR signal over the 10 min period mimicking the fMRI study without the presentation of stimulus animals in the vivarium did not differ by more than 2% for any of the four animals studied (Fig 13). These data corroborate our previous work reporting a baseline variability in MR signal of ca 2% in fully conscious animals imaged with an INSL rodent restrainer system and dual coil RF electronics at 4.7T [136,137,144]. These data also show that drift is not an issue in the present study using an fMRI scanning protocol of 10 min in duration. However, it should be noted, that in our hands using the same technology, fMRI scanning protocols that exceed 20 min in duration show drift.

### Controlling for vascular responsivity

V1a receptors are found on arterial smooth muscle and mediate vasopressin-induced vasoconstriction. Since SRX251 is a selective, long acting V1a antagonist it would be expected to block these peripheral vascular receptors in addition to those localized to neurons in the CNS. While vasopressin is not involved in the moment-to-moment regulation of vascular tone and baseline blood pressure [184], there is always the possibility that SRX251 can affect vascular responsivity and the BOLD signal. To control for this, male rats were treated with a 5 mg/kg oral dose of SRX251 (n = 4) or water vehicle (n = 4). Ninety min later animals were imaged as described above. During a 10 min imaging session animals were exposed to 10% carbon dioxide using an off-on, box-car design of 2 min intervals. There was no ostensible difference between vehicle and SRX251 treatment in vascular responsivity to the direct vasodilatory effects of carbon dioxide (Fig 10). Both treatments showed a similar time course of BOLD activation and recovery with signal changes exceeding 6%. The average BOLD activation map for each treatment was also very similar with the cortex and thalamus showing robust signal change in response to carbon dioxide. These same carbon dioxide challenge studies were run for animals treated with 5 mg/kg fluoxetine (Fig 10) and showed no apparent affect on vascular responsivity with blockade of the serotonin transporter. Interestingly, the activation maps for the three experimental conditions show intense signal change over the dorsal thalamus. Vascular casts of coronal sections of rat brain show a high density of small arterial blood vessels in this area [185].

### Assessing motion artifact

Subject motion is an important issue in fMRI data analysis; even the slightest movement during the scan can displace voxel location corresponding to a distinct physical area. Unlike human fMRI, this issue is more prevalent in small animals like rats as voxel size is much larger than physical (anatomical) area in the brain. The change in signal intensity due to motion can be (and usually is) greater than BOLD signal especially at the edge of the brain and tissue boundaries which essentially leads to artifact in the activation map. To avoid this, "motion correction" has become common preprocessing step in fMRI data analysis. Commonly used motion correction tools include AIR [186-188], *AFNI *[189], and statistical parametric mapping (SPM) realign tools [190].

However, it has been reported that motion correction may induce spurious activation in motion-free fMRI data [191]. This artifact stems from the fact that activated areas behave like biasing outliers for the difference of square-based measures usually driving such registration methods. This problem is amplified in case of small animals where the BOLD signal change can be 10% or greater over baseline. Indeed, if motion parameters are included in the general linear model for event-related data, it makes little difference if motion correction is actually applied to the data [192].

The experiments conducted in this work are a single epoch event-related design. To assess false activation due to subject motion we collected fMRI data from awake rats (n = 8) over a 10 min scanning session in the absence of any stimulation. From these empirical data, a series of virtual fMRI data were numerically generated using a tri-linear interpolation algorithm with Gaussian noise and a preset amount of rigid body motion in random direction. The amount of motion introduced was in increment of 1/10 of a voxel (ca 50 μm) up to 1 voxel (486 μm). The data were analyzed with statistical *t *tests on each voxel for each subject within their original coordinate system. The control window was the first 50 time periods (5 min) where as the stimulation window was the remaining 50 time periods (5 min) as described for the fMRI studies above. The *t *test statistics used a 95% confidence level, two-tailed distributions, and heteroscedastic variance assumptions. In this case a multiple comparison control (false detection rate) was *not *used to avoid suppression of any spurious activation. There is no significant change in BOLD signal or the number of activated voxels up to ca 300 μm (or 6/10 of voxel) motion. Both, number of voxels and percent BOLD signal, increases dramatically as it approaches one voxel of motion.

**Figure 14:**
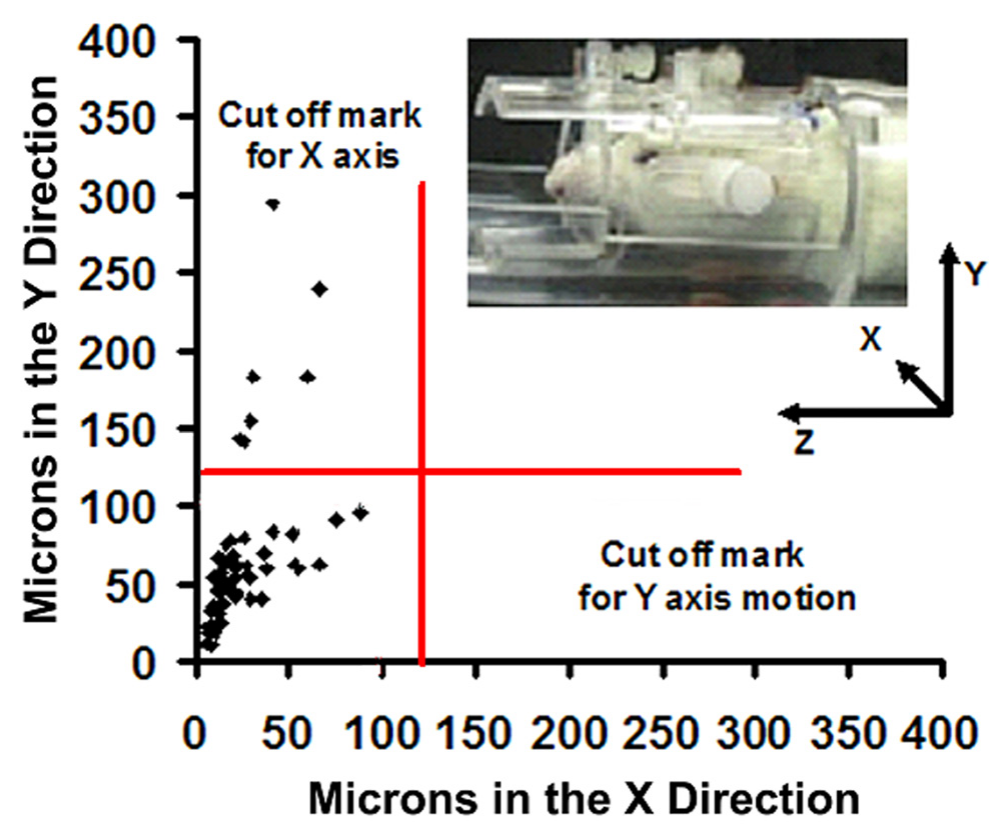
**Plotting subjects that meet experimental criteria for acceptable voxel movement**. Shown are motion data in the X Y directions for 71 animals. Data points within the red lines marked at 120 μm were judged to be acceptable. Note that unacceptable motion was limited to the Y direction (movement of the head up-and-down near the neck). The insert shows a rat positioned in the head restrainer.

For each subject (n = 71) in this work, rigid body motion in x, y and z direction was computed with Stimulate [193] software using center of intensity method. Standard deviation of this data gives measure of how widely spread the motion is for each subject. We set conservative criteria of 120 μm standard deviation of motion in any direction as acceptance criteria. In these experiments, motion in the z and x direction was small as compared to y direction. Animals showing an average displacement exceeding 25% of the total inplane (X-Y) voxel resolution (> 120 μm out of 468 μm) or more than 25% displacement in the slice (Z) direction (> 300 μm out of 1200 μm slice thickness) were excluded. Most of the motion was in y-direction (64 μm ± 42 μm) and can be attributed to limitations in the design of the rat head holder. A plot of the x-y-z spread of the standard deviation of the 71 animals tested showed 9 animals had motion greater that defined criteria and were excluded from data analysis (Fig 14). The remaining 62 animals fell under the acceptance criteria and were included in the study without any motion correction.

### Data analysis

Anatomy images for each subject were obtained at a resolution of 256^2 ^× 12 slices and a FOV of 30 mm with a slice thickness of 1.2 mm. Subsequent functional imaging was performed at a resolution of 64^2 ^× 12 slices with the same FOV and slice thickness. Each subject was registered to a segmented rat brain atlas. The alignment process was facilitated by an interactive graphic user interface. The affine registration involved translation, rotation, and scaling in all three dimensions, independently. The matrices that transformed the subject's anatomy to the atlas space were used to embed each slice within the atlas. All transformed pixel locations of the anatomy images were tagged with the segmented atlas major and minor regions creating a fully segmented representation of each subject. The inverse transformation matrix [*T*_*i*_]^-1^for each subject (*i*) was also calculated.

The fully segmented rat brain atlas has the potential to delineate and analyze more than 1200 distinct anatomical volumes within the brain. Because the in-plane spatial resolution of our functional scans (data matrix, 64 × 64; FOV 3.0 cm) is 486 μm^2 ^with a depth of 1200 μm, many small brain areas (e.g. nucleus of the lateral olfactory tract) cannot be resolved; or, if they could be resolved they would be represented by one or two voxels only (e.g. arcuate nucleus of the hypothalamus). Consequently, small detailed regions are not included in the analysis or are grouped into larger "minor volumes" of similar anatomical classification. For example, in these studies we have the basal nucleus of the amygdala listed as a minor volume. This area is a composition of the basomedial anterior part, basomedial posterior part, basolateral anterior part and basolateral posterior part with a composite voxel size of 54. In this study, 12 brain slices were collected extending from the tip of the forebrain to the end of the cerebrum stopping at the midbrain just rostral to the cerebellum. Within these rostral/caudal boundaries we delineated 83 minor volumes. In addition, we grouped brain areas into "major volumes" (e.g., amygdala, hippocampus, hypothalamus, and cerebrum). The volume of activation (number of significant voxels) can be visualized in these 3D major and minor anatomical groupings (Fig 1). We also combined minor volumes to form functional neuroanatomical pathways as shown in Fig 2.

Each scanning session consisted of 100 data acquisitions with a period of 6 sec each for a total lapse time of 600 sec or 10 min. The control window was the first 50 scan repetitions while the stimulation window was 51–100 scans post-stimulation period. Statistical *t *tests were performed on each voxel (4,800 in number) of each subject within their original coordinate system. The baseline threshold was set at 2%. The *t *test statistics used a 95% confidence level, two-tailed distributions, and heteroscedastic variance assumptions. As a result of the multiple *t *test analyses performed, a false-positive detection controlling mechanism was introduced [194]. This subsequent filter guaranteed that, on average, the false-positive detection rate was below our cutoff of 0.05. The formulation of the filter satisfied the following expression:

$$P_{\left(i\right)}\leq \frac{i}{V}\frac{q}{c(V)}$$

where *P*_(*i*) _is the *p *value based on the *t *test analysis. Each pixel (*i*) within the region of interest (ROI) containing (*V*) pixels was ranked based on its probability value. The false-positive filter value *q *was set to be 0.05 for our analyses, and the predetermined constant *c*(*V*) was set to unity, which is appropriate for data containing Gaussian noise such as fMRI data [194]. These analysis settings provided conservative estimates for significance. Those pixels deemed statistically significant retained their percentage change values (stimulation mean minus control mean) relative to control mean. All other pixel values were set to zero.

A statistical composite was created for each group of subjects. The individual analyses were summed within groups. The composite statistics were built using the inverse transformation matrices. Each composite pixel location (i.e., row, column, and slice), premultiplied by [*T*_*i*_]^-1^, mapped it within a voxel of subject (*i*). A tri-linear interpolation of the subject's voxel values (percentage change) determined the statistical contribution of subject (*i*) to the composite (row, column, and slice) location. The use of [*T*_*i*_]^-1 ^ensured that the full volume set of the composite was populated with subject contributions. The average value from all subjects within the group determined the composite value. The BOLD response maps of the composite were somewhat broader in their spatial coverage than in an individual subject; so only average number of activated pixels that has highest composite percent change values in particular ROI was displayed in composite map. Activated composite pixels are calculated as follows:

$$Activated\;Composite\;Pixels\;ROI(j)=\frac{\sum_{i=1}^{N}Activated\;Pixels\;Subject(i)\;ROI(j)}{N}$$

The composite percent change for the time history graphs for each region was based on the weighted average of each subject, as follows:

$$Composite\;Percent\;Change=\frac{\sum_{i=1}^{N}Activated\;Pixel\;Subject(i)\times Percent\;Change(i)}{Activated\;Composite\;Pixels}$$

where *N *is number of subjects.

The percent change in BOLD signal for time history graphs (see Figs 6, 7, 8) were only calculated as an average of the positive voxels within a volume of interest and did not include negative voxels. To average both positive and negative voxels assumes that a volume of interest, e.g. the somatosensory cortex, is functionally homogenous, i.e. all cortical columns do the same thing and their combined activity (percent change in BOLD signal) reflects a common metabolic change in response to a stimulus. Evidence would suggest that anatomically delineated brain areas as shown in different 2D digitized atlases and our own 3D atlas may not be functionally homogenous [195,196]. There is a growing body of literature from humans and animals using multiple imaging techniques that excitatory and inhibitory inputs to the cortex produce "surround inhibition" around the principle cortical column receiving direct thalamic input. The hemodynamic response to this well recognized cortical event is a decrease in blood oxygenation and blood volume extending over 1–2 mm [197-202]. The voxels in the area of "surround inhibition" displaying negative BOLD do not reflect neurons engaged in a network response to the activation of a central cortical column; instead, this is a passive and active event of arteriole vasoconstriction shunting blood to the active column [197]. For this reason we chose to eliminate averaging negative BOLD and positive BOLD voxels in a volume of interest. To do so, we believe reduces and masks the true change in active BOLD signal.

## Abbreviations

ACA: anterior cingulated; ACB: accumbens; AH: anterior hypothalamus; AL: agranular insular cortex; ALP: anterior lobe pituitary; AM: anteromedial nucleus thalamus; AON: anterior olfactory nucleus; AUD: auditory cortex; AV: anteroventral nucleus thalamus; BNST: bed nucleus stria terminalis; CA1: hippocampus; CA3: hippocampus; COA: cortical nucleus amygdale; DG: dentate gyrus; DS: dorsal striatum; ENT: entorhinal cortex; IC: inferior colliculi; ILA: infralimbic cortex; IPN: interpeduncular nucleus; LHA: lateral hypothalamus; LP: lateral posterior nucleus thalamus; LS: lateral septum; MEA: medial amygdale; MM: mammillary nuclei; MO: motor cortex; MRN: mesencephalic reticular nucleus; ORB: orbital cortex; OT: olfactory tubercle; PAG: periaqueductal gray; PIR: piriform cortex; PL: prelimbic cortex; PTL: parietal cortex; PVN: paraventricular nucleus hypothalamus; PVT: paraventricular nucleus thalamus; RE nucleus reunions thalamus; RSP: retrosplenial cortex; RPH: raphe; SC: superior colliculi; SN: substantia nigra; SSP: primary somatosensory cortex; VMH: ventromedial hypothalamus; VP: ventral posterior nucleus thalamus; VTA: ventral tegmental area

## Competing interests

Craig Ferris and Mathew Brevard have a financial interest in Insight MRI the bioengineering company that manufactures the animal imaging equipment used in these studies. Craig Ferris and Neal Simon have a financial interest in Azevan Pharmaceuticals the company developing SRX251 an orally active V1a receptor antagonist used in these studies.

## Authors' contributions

Each author has made a significant contribution to these studies either in the acquisition of data (TM, MF, MB), software development, data analysis (PK, MM), or study design and manuscript preparation (CF, RB, CB, NS). All authors read and approved the final manuscript.

## References

[B1] Huntingford FA, Turner AK (1987). Animal Conflict.

[B2] Potter LB, Mercy JA, Stoff DM, J B, Maser JD (1997). Public health perspective on interpersonal violence among youths in the United States. Handbook of Antisocial Behavior.

[B3] Cloninger CR, Bayon C, Przybeck TR, Stoff DM, Breiling J, Maser JD (1997). Epidemiology and Axis I comorbidity of antisocial personality. Handbook of Antisocial Behavior.

[B4] Connor DF (2002). Aggression and Antisocial Behavior in Children and Adolescents: Research and Treatment.

[B5] Nagin D, Tremblay RE (1999). Trajectories of boy's physical aggression, opposition, and hyperactivity on the path to physically violent and nonviolent juvenile delinquency. Child Dev.

[B6] Tremblay RE, Nagin DS, Sequin JR, Zoccolillo M, Zelazo PD, Boivin M, Perusse D, Japel C (2005). Physical aggression during early childhood: trajectories and predictors. Can Child Adolesc Psychiatr Rev.

[B7] Dodge KA, Peplerr DJ, Rubin KH (1991). The structure and function of reactive and proactive aggression. The Development and Treatment of Childhood Aggression.

[B8] Kazdin AE, Bass D, Siegel T, Thomas C (1989). Cognitive-behavioral therapy and relationship therapy in the treatment of children referred for antisocial behavior. J Consult Clin Psychol.

[B9] Malone RP, Luebbert JF, Delaney MA, Biesecker KA, Blaney BL, Rowan AB, Campbell M (1997). Nonpharmacological response in hospitalized children with conduct disorder. J Am Acad Child Adolesc Psychiatry.

[B10] Steiner H, Saxene K, Chang K (2003). Psychopharmacological strategies for the treatment of aggression in youth. CNS Spectrums.

[B11] Olivier B, Mos J (1990). Serenics, serotonin and aggression. Prog Clin Biol Res.

[B12] Olivier B, Mos J, Hartog J, Rasmussen DL (1990). Serenics: a new class of drugs for putative selective treatment of pathological destructive behavior. Drug News Perspective.

[B13] Stanislav SW, Fabre T, Crismon ML, Childs A (1994). Buspirone's efficacy in organic-induced aggression. J Clin Psychopharmacol.

[B14] Coccaro EF, Kavoussi RJ (1997). Fluoxetine and impulsive aggressive behavior in personality-disordered subjects. Arch Gen Psychiatry.

[B15] Pfeffer CR, Jiang H, Domeshek LJ (1997). Buspirone treatment of psychiatrically hospitalized prepubertal children with symptoms of anxiety and moderately severe aggression. J Child Adolesc Psychopharmacol.

[B16] Ratey J, Sovner R, Parks A, Rogentine K (1991). Buspirone treatment of aggression and anxiety in mentally retarded patients: a multiple-baseline, placebo lead-in study. J Clin Psychiatry.

[B17] Ricketts RW, Goza AB, Ellis CR, Singh YN, Chambers S, Singh NN, Cooke JC (1994). Clinical effects of buspirone on intractable self-injury in adults with mental retardation. J Am Acad Child Adolesc Psychiatry.

[B18] Ferris CF, Bock G, Goode J (2005). Vasopressin/oxytocin and aggression. Novartis Found Symp.

[B19] Guillon CD, Koppel GA, Brownstein MJ, Chaney MO, Ferris CF, Lu S-F, Fabio KM, Miller MJ, Heindel ND, Hunden DC (2007). Azetidinones as vasopressin V1a antagonists. Bioorg Med Chem.

[B20] Ferris CF, Lu S-F, Messenger T, Guillon CD, Koppel GA, Miller MJ, Heindel ND, Simon NG (2006). Orally active vasopressin V1a receptor antagonist, SRX251, selectively blocks aggressive behavior. Pharmacol Biochem Behav.

[B21] Delgado JMR, Valzelli I, Morgese I (1980). Neural constellations in aggressive behavior. Aggression and Violence: A Psychobiological and Clinical Approach.

[B22] Ferris CF, Nelson RJ (2006). Neuroplasticity and aggression: an interaction between vasopressin and serotonin. Biology of Aggression.

[B23] Moyer KE (1968). Kinds of aggression and their physiological basis. Comm Behav Biol.

[B24] Adams DB (1979). Brain mechanisms for offensive, defense and submission. Behav Brain Sci.

[B25] Albert DJ, Walsh ML (1984). Neural systems and the inhibitory modulation of agonistic behavior: a comparison of mammalian species. Neurosci Biobehav Rev.

[B26] Ricci LA, Grimes JM, Melloni RH (2006). Lasting changes in neuronal activation patterns in select forebrain regions of aggressive, adolescent anabolic/androgenic steroid-treated hamsters. Behav Brain Res.

[B27] Hasen SN, Gammie SC (2005). Differential fos activation in virgin and lactating mice in response to an intruder. Physiol Behav.

[B28] Potegal M, Ferris CF, Hebert M, Meyerhoff J, Skaredoff L (1996). Attack priming in female Syrian golden hamsters is associated with a c-fos coupled process within the corticomedial amygdala. Neuroscience.

[B29] Delville Y, De Vries GJ, Ferris CF (2000). Neural connections of the anterior hypothalamus and agonistic behavior in golden hamsters. Brain Behav Evol.

[B30] Haller J, Toth M, Halasz J, De Boer SF (2006). Patterns of violent aggression-induced brain c-fos expression in male mice selected for aggressiveness. Physiol Behav.

[B31] Halasz J, Toth M, Kallo I, Liposits Z, Haller J (2006). The activation of prefrontal cortical neurons in aggression – A double labeling study. Behav Brain Res.

[B32] Knyshevski I, Connor DF, Harrison RJ, Ricci LA, Melloni RHJ (2005). Persistent activation of select forebrain regions in aggressive, adolescent cocaine-treated hamsters. Behav Brain Res.

[B33] Blanchard RJ, Blanchard DC (1977). Aggressive behavior in the rat. Physiol Behav.

[B34] Ferris CF, Febo M, Luo F, Schmidt K, Brevard ME, Kulkarni P, Messenger TL, Harder JA, King JA (2006). Functional magnetic resonance imaging in conscious animals: A new tool in behavioral neuroscience research. Journal of Neuroendocrinology.

[B35] Ogawa S, Lee TM, Nayak AS, Glynn P (1990). Oxygenation-sensitive contrast in magnetic resonance image of rodent brain at high magnetic fields. Magn Reson Med.

[B36] Sokolloff L, Reivich M, Kennedy C, Des Rosiers MH, Patlak CS, Pettigrew KD, Sakurada O, Shinohara M (1977). The [14C] deoxyglucose method for the measurement of local cerebral glucose utilization: theory, procedure, and normal values in the conscious and anesthetized albino rat. J Neurochem.

[B37] Fox PT, Raichle ME (1986). Focal physiological uncoupling of cerebral blood flow and oxidative metabolism during somatosensory stimulation in human subjects. Proc Natl Acad Sci USA.

[B38] Ramsey NF, Kirkby BS, Van Gelderen P, Berman KF, Duyn JH, Frank JA, Mattay VS, Van Horn JD, Esposito G, Moonen CT (1996). Functional mapping of human sensorimotor cortex with 3D BOLD fMRI correlates highly with H2(15)O PET rCBF. J Cereb Blood Flow Metab.

[B39] Belliveau JW, Kennedy DN, McKinstry RC, Buchbinder BR, Weisskoff RM, Cohen MS, Vevea JM, Brady TJ, Rosen BR (1990). Functional mapping of the human visual cortex by magnetic resonance imaging. Science.

[B40] Logothetis NK, Pauls J, Augath M, Trinath T, Oeltermann A (2001). Neurophysiological investigation of the basis of the fMRI signal. Nature.

[B41] Smith AJ, Blumenfeld H, Behar KL, Rothman DL, Shulman RG, F H (2002). Cerebral energetics and spiking frequency: The neurophysiological basis of fMRI. Proc Natl Acad Sci USA.

[B42] Karli P, Vergnes M, Didiergeorges F, Sigg E, Garattini S (1969). Rat-mouse interspecific aggressive behavior and its manipulation by brain ablation and by brain stimulation. Biology of Aggressive Behavior.

[B43] Hess WR, Brugger M (1943). Das subkortikale Zentrum der affektiven Abwehrreacktion. Helvetica physiologica et pharacologica Acta.

[B44] Roberts W, Steinberg M, Means L (1967). Hypothalamic mechanisms for sexual, aggressive, and other motivational behaviors in the opossum, *Didelphis Virginiana*. J Comp Physiol Psychol.

[B45] Robinson B, Alexander M, Bowne G (1969). Dominance reversal resulting from aggressive response evoked by brain telestimulation. Physiol Behav.

[B46] Wasman M, Flynn JP (1962). Directed attack elicited from the hypothalamus. Arch Neurol.

[B47] Adams DB (1971). Defense and territorial behaviour dissociated by hypothalamic lesions in the rat. Nature.

[B48] Panksepp J (1971). Effects of hypothalamic lesions on mouse-killing and shock-induced fighting in rats. Physiol Behav.

[B49] Roeling T, Veening J, Kruk M, Peters J, Vermelis M, Nieuwenhuys R (1994). Efferent connections of the hypothalamic "aggressive area" in the rat. Neuroscience.

[B50] Nieuwenhuys R, Geeraedts LM, Veening JG (1982). The medial forebrain bundle of the rat. I. General introduction. J Comp Neurol.

[B51] Goto M, Canteras NS, Burns G, Swanson LW (2005). Projection from the subfornical region of the lateral hypothalmaic area. J Comp Neurol.

[B52] Berk ML, Finkelstein JA (1982). Efferent connections of the lateral hypothalamic area of the rat: an autoradiographic investigation. Brain Res Bull.

[B53] Antonucci AS, Gansler DA, Tan S, Bhadelia R, Patz S, Fulwiler C (2006). Orbitofrontal correlates of aggression and impulsivity in psychiatric patients. Psychiatry Res.

[B54] Lotze M, Veit R, Anders S, Birbaumer N (2007). Evidence for a different role of the ventral and dorsal medial prefrontal cortex for social reactive aggression: An interactive fMRI study. NeuroImage.

[B55] Nomura M, Nomura Y (2006). Psychological, neuroimaging, and biochemical studies on functional association between impulsive behavior and the 5-HT2A receptor gene polymorphism in humans. Ann N Y Acad Sci.

[B56] Keay KA, Bandler R (2001). Parallel circuits mediating distinct emotional coping reactions to different types of stress. Neurosci Biobehav Rev.

[B57] Machado CJ, Bachevalier J (2006). The impact of selective amygdala, orbital frontal cortex, or hippocampal formation lesions on established social relationships in rhesus monkeys (Macaca mulatta). Behav Neurosci.

[B58] Raine A (2002). Annotation: the role of prefrontal deficits, low autonomic arousal, and early health factors in the development of antisocial and aggressive behavior in children. J Child Psychol Psychiatry.

[B59] Blanchard RJ, Fukunaga K, Blanchard DC, Kelley MJ (1975). Conspecific aggression in the laboratory rat. J Comp Physiol Psychol.

[B60] Vanderwolf CH (1978). Behavior of the rat after removal of the neocortex and hippocampal formation. J Comp Physiol Psychol.

[B61] Mitchell AS, Dalrymple-Alford JC, Christie MA (2002). Spatial working memory and the brainstem cholinergic innervation to the anterior thalamus. J Neurosci.

[B62] Wolff M, Gibb SJ, Dalrymple-Alford JC (2006). Beyond spatial memory: the anterior thalamus and memory for the temporal order of a sequence of odor cues. J Neurosci.

[B63] Jenkins TA, Dias R, Amin E, Aggleton JP (2002). Changes in Fos expression in the rat brain after unilateral lesions of the anterior thalamic nuclei. Eur J Neurosci.

[B64] Hamani C, Ewerton FI, Boniha SM, Ballester G, Mello LE, Lozano AM (2004). Bilateral anterior thalamic nucleus lesions and high-frequency stimulation are protective against pilocarpine-induced seizures and status epilepticus. Neurosurgery.

[B65] Miller JW, McKeon C, Ferrendelli JA (1987). Functional anatomy of pentylenetetrazol and electroshock seizures in the rat brainstem. Annals of Neurology.

[B66] Mirski MA, Rossell LA, Terry JB, Fisher RS (1997). Anticonvulsant effect of anterior thalamic high frequency electrical stimulation in the rat. Epilepsy Res.

[B67] Brevard M, Kulkarni P, King JA, Ferris CF (2006). Imaging the neural substrates involved in the genesis of generalized clonic seizures. Epilepsia.

[B68] Mirski MA, Tsai YC, Rossell LA, Thakor NV, Sherman DL (2003). Anterior thalamic mediation of experimental seizures: selective EEG spectral coherence. Epilepsia.

[B69] Hodaie M, Wennberg RA, Dostrovsky JO, Lozano AM (2002). Chronic anterior thalamus stimulation for intractable epilepsy. Epilepsia.

[B70] Kerrigan JF, Litt B, Fisher RS, Cranstoun S, French JA, Blum DE, Dichterr M, Shetter A, Baltuch G, Jaqqi J (2004). Electrical stimulation of the anterior nucleus of the thalamus for the treatment of intractable epilepsy. Epilepsia.

[B71] Cannon WB (1927). The James-Lang theory of emotion: A critical examination and an alternative theory. Am J Psychol.

[B72] Papez JW (1937). A proposed mechanism of emotion. Arch Neurol Psychiatry.

[B73] Sikes RW, Chronister RB, White LE (1977). Origin of the direct hippocampus-anterior thalamic bundle in the rat: A combined horseradish peroxidase-Golgi analysis. Exp Neurol.

[B74] Swanson LW, Cowan WM (1977). An autoradiographic study of the organization of the efferent connections of the hippocampal formation in the rat. J Comp Neurol.

[B75] Shibata H (1992). Topographic organization of subcortical projections to the anterior thalamic nuclei in the rat. J Comp Neurol.

[B76] Seki M, Zyo K (1984). Anterior thalamic afferents from the mamillary body and the limbic cortex in the rat. J Comp Neurol.

[B77] Thompson SM, Robertson RT (1987). Organization of subcortical pathways for sensory projections to the limbic cortex I. Subcortical projections to the medial limbic cortex in the rat. J Comp Neurol.

[B78] Domesick VB (1972). Thalamic relationship of the medial cortex in the rat. Brain Behav Evol.

[B79] Shibata H (1993). Efferent projections from the anterior thalamic nuclei to the cingulate cortex in the rat. J Comp Neurol.

[B80] Sripanidkulchai K, Wyss JM (1986). Thalamic projections to retrosplenial cortex in the rat. J Comp Neurol.

[B81] Ferris CF, Potegal M (1988). Vasopressin receptor blockade in the anterior hypothalamus suppresses aggression in hamsters. Physiol Behav.

[B82] Potegal M, Ferris CF (1990). Intraspecific aggression in male hamsters is inhibited by intrahypothalamic vasopressin-receptor antagonist. Agg Behav.

[B83] Young L, Winslow J, Nilsen R, Insel T (1997). Species differences in V1a receptor gene expression in monogamous and nonmonogamous voles: behavioral consequenses. Behav Neurosci.

[B84] Caldwell HK, Albers HE (2004). Effect of photoperiod on vasopressin-induced aggression in Syrian hamsters. Horm Behav.

[B85] Harrison RJ, Connor DF, Nowak C, Nash K, Melloni RH (2000). Chronic anabolic-androgenic steriod treatment during adolescence increases anterior hypothalamic vasopressin and aggression in intact hamsters. Psychoneuroendocrinology.

[B86] Haller J, Makara GB, Barna I, Kovacs K, Nagy J, Vecsernyes M (1996). Compression of the pituitary stalk elicits chroninc increases in CSF vasopressin, oxytocin as well as in social investigation and aggressiveness. J Neuroendocrinol.

[B87] Coccaro EF, Kavoussi RJ, Hauger RL, Cooper TB, Ferris CF (1998). Cerebrospinal fluid vasopressin levels: correlates with aggression and serotonin function in personality-disordered subjects. Arch Gen Psychiatry.

[B88] Thompson RR, George K, Walton JC, Orr SP, Benson J (2006). Sex-specific influences of vasopressin on human social communication. Proc Natl Acad Sci USA.

[B89] Thompson R, Gupta S, Miller K, Mills S, Orr S (2004). The effects of vasopressin on human facial responses related to social communication. Psychoneuroendocrinology.

[B90] Delville Y, Melloni RH, Ferris CF (1998). Behavioral and neurobiological consequences of social subjugation during puberty in golden hamsters. J Neurosci.

[B91] Veenema AH, Blume A, Niederle D, Buwalda B, Neumann ID (2006). Effects of early life stress on adult male aggression and hypothalamic vasopressin and serotonin. Eur J Neurosci.

[B92] DeLeon KR, Grimes JM, Melloni RH (2002). Repeated anabolic-androgenic steroid treatment during adolescence increases vasopressin V(1A) receptor binding in Syrian hamsters: correlation with offensive aggression. Horm Behav.

[B93] Jackson D, Burns R, Trksak G, Simeone B, Deleon KR, Connor DF, Harrison RJ, Melloni RH (2005). Anterior hypothalamic vasopressin modulates the aggression-stimulating effects of adolescent cocaine exposure in Syrian hamsters. Neuroscience.

[B94] Ferris CF, Axelson JF, Martin AM, Roberge LF (1989). Vasopressin immunoreactivity in the anterior hypothalamus is altered during the establishment of dominant/subordinate relationships between hamsters. Neuroscience.

[B95] Cooper MA, Karom M, Huhman KL, Albers HE (2005). Repeated agonistic encounters in hamsters modulate AVP V1a receptor binding. Horm Behav.

[B96] Bester-Meredith J, Young L, Marker C (1999). Species differences in paternal behavior and aggression in peromyscus and their associations with vasopressin immunoreactivity and receptors. Horm Behav.

[B97] Bester-Meredith JK, Marker CA (2001). Vasopressin and aggression in cross-fostered California mice (Peromyscus californicus) and white-footed mice (Peromyscus leucopus). Horm Behav.

[B98] Hull EM, Micevych P, Hammer R (1995). Dopaminergic influences on male rat sexual behavior. Neurobiological effects of sex steriod hormones.

[B99] Ferris CF, Delville Y, Grzonka Z, Luber-Narod J, Insel TR (1993). An iodinated vasopressin (V1) antagonist blocks flank marking and selectively labels neural binding sites in golden hamsters. Physiol Behav.

[B100] Barbeis C, Balestre MN, Jard S, Tribollet E, Arsenijevic Y, Dreifuss JJ, Bankowski K, Manning M, Chan WY, Schlosser SS (1995). Characterization of a novel, linear radioiodinated vasopressin antagonist: an excellent radioligand for vasopressin V1a receptors. Neuroendocrinology.

[B101] Tribollet E, Barberis C, Jard S, Dubois-Dauphin M, Dreifuss JJ (1988). Localization and pharmacological characterization of high affinity binding sites for vasopressin and oxytocin in the rat brain by light microscopic autoradiography. Brain Res.

[B102] Insel TR, Wang Z, Ferris CF (1994). Patterns of brain vasopressin receptor distribution associated with social organization in microtine rodents. J Neurosci.

[B103] Young LJ, Toloczko D, Insel TR (1999). Localization of vasopressin (V1a) receptor binding and mRNA in rhesus monkey brain. J Neuroendocrinol.

[B104] Mann JJ, Brent DA, Arango V (2001). The neurobiology and genetics of suicide and attempted suicide: A focus on the serotonergic system. Neuropsychopharmacology.

[B105] Manuck SB, Kaplan JR, Lotrich FE, Nelson RJ (2006). Brain serotonin and aggressive disposition in humans and nonhuman primates. Biology of Aggression.

[B106] Zubieta JA, Alessi NE (1992). Acute and chronic administration of trazodone in the treatment of disruptive behavior disorders in children. J Clin Psychopharmacol.

[B107] Coccaro EF, Astill JL, Herbert JL, Schut AG (1990). Fluoxetine treatment of impulsive aggression in DSM-III-R personality disorder patients. Journal of Clinical Psychopharmacology.

[B108] Cherek DR, Lane SD (2001). Acute effects of D-fenfluramine on simultaneous measures of aggressive escape and impulsive responses of adult males with and without a history of conduct disorder. Psychopharmacology (Berl).

[B109] Cherek D, Lane S, Pietras C, Steinberg J (2002). Effects of chronic paroxetineadministration on measures of aggressive and impulsive responses of adult males with a history of conduct disorder. Psychopharmacology (Berl).

[B110] Barnes NM, Sharp T (1999). A review of central 5-HT receptors and their function. Neuropharmacol.

[B111] Le Poul E, Laaris N, Doucet E, Laporte A, Hamon M, Lanfumey L (1995). Early desensitization of somato-dendritic 5-HT1A autoreceptors in rats treated with fluoxetine and paroxetine. Naunyn Schmiedebergs Arch Pharmacol.

[B112] Elena Castro M, Diaz A, del Olmo E, Pazos A (2003). Chronic fluoxetine induces opposite changes in G protein coupling at pre and postsynaptic 5-HT1A receptors in rat brain. Neuropharmacology.

[B113] Pejchal T, Foley MA, Kosofsky BE, Waeber C (2002). Chronic fluoxetine treatment selectively uncouples raphe 5-HT(1A) receptors as measured by [(35)S]-GTP gamma S autoradiography. Br J Pharmacol.

[B114] Blier P, de Montigny C (1994). Current advances in the treatment of depression. Trends Pharmacol Sci.

[B115] Jacobs BL, Azmitia EC (1992). Structure and function of the brain serotonin system. Physiol Rev.

[B116] Ferris CF, Ferris C, Grisso T (1996). Serotonin inhibits vasopressin facilitated aggression in the Syrian hamster. Understanding aggressive behavior in children.

[B117] Lorrain DS, Riolo JV, Matuszewich L, Hull EM (1999). Lateral hypothalamic serotonin inhibits nucleus accumbens dopamine: implications for sexual satiety. J Neurosci.

[B118] Rosen RC, Lane SDRM, Menza M (1999). Effects of SSRIs on sexual function: a critical review. J Clin Psychopharmacol.

[B119] Damsa C, Bumb A, Bianchi-Demicheli F, Vidailhet P, Sterck R, Andreoli A, Beyenburg S (2004). "Dopamine-dependent" side effects of selective serotonin reuptake inhibitors: a clinical review. J Clin Psychiatry.

[B120] Landgraf R, Gerstberger R, Montkowski A, Probst JC, Wotjak CT, Holsboer Fea (1995). V1 vasopressin receptor antisense oligodeoxynucleotide into septum reduces vasopressin binding, social discrimination abilities, and anxiety-related behavior in rats. J Neurosci.

[B121] Liebsch G, Wotjak CT, Landgraf R, Engelmann M (1996). Septal vasopressin modulates anxiety-related behaviour in rats. Neurosci Lett 217: 101–104. Neurosci Lett.

[B122] Wigger A, Sanchez MM, Mathys KC, Ebner K, Frank E, Liu D, Kresse A, Neumann ID, Holsboer F, Plotsky PM (2004). Alterations in central neuropeptide expression, release, and receptor binding in rats bred for high anxiety: critical role of vasopressin. Neuropsychopharmacology.

[B123] Ferris CF, Rasmussen MF, Messenger TL, Koppel GA (2001). Vasopressin-dependent flank marking in golden hamsters is suppressed by drugs used in the treatment of obsessive-compulsive disorder. BMC Neuroscience.

[B124] Hirano K, Kimura R, Sugimoto Y, Yamada J, Uchida S, Kato Y, Hashimoto H, Yamada S (2005). Relationship between brain serotonin transporter binding, plasma concentration and behavioural effect of selective serotonin reuptake inhibitors. Br J Pharmacol.

[B125] Leveleki C, Sziray N, Levay G, Barsvari B, Soproni K, Mikics E, Haller J (2006). Pharmacological evaluation of the stress-induced social avoidance model of anxiety. Brain Res Bull.

[B126] Gsell W, Burke M, Wiedermann D, Bonvento G, Silva AC, Dauphin F, Buhrle C, Hoehn M, Schwindt W (2006). Differential effects of NMDA and AMPA glutamate receptors on functional magnetic resonance imaging signal and evoked neuronal activity during forepaw stimulation of the rat. J Neurosci.

[B127] Lehman MN, Adams DR (1977). A statistical and motivational analysis of the social behaviours of the male laboratory rat. Behaviour.

[B128] Albert DJ, Walsh ML, Gorzalka BB, Siemiens Y, Louie H (1986). Testosterone removal in rats results in decrease in social aggression and a loss of social dominance. Physiol Behav.

[B129] Blanchard DC, Takahashi SN (1988). No change in intermale aggression after amygdala lesions which reduce freezing. Physiol Behav.

[B130] Albert DJ, Walsh ML (1982). Medial hypothalamic lesions in the rat enhance reactivity and mouse killing but not social aggression. Physiol Behav.

[B131] Zhang Z, Andersen AH, Avison MJ, Gerhardt GA, Gash DM (2000). Functional MRI of apomorphine activation of the basal ganglia in awake rhesus monkeys. Brain Res.

[B132] King JA, Garelick TS, Brevard ME, Chen W, Messenger TL, Duong TQ, Ferris CF (2005). Procedure for minimizing stress for fMRI studies in conscious rats. J Neurosci Methods.

[B133] Ferris CF, Snowdon CT, King JA, Sullivan JM, Ziegler TE, Olson DP, Schultz-Darken NJ, Tannenbaum PL, Ludwig R, Wu Z (2004). Activation of neural pathways associated with sexual arousal in non-human primates. J Magn Reson Imaging.

[B134] Tenney JR, Duong TQ, King JA, Ludwig R, Ferris CF (2003). Corticothalamic modulation during absence seizures:A functional MRI approach. Epilepsia.

[B135] Tenney JR, Brevard ME, King JA, Ferris CF (2004). fMRI of generalized absence seizures in conscious marmoset monkeys reveals corticothalamic activation. Epilepsia.

[B136] Febo M, Segarra A, Nair G, Schmidt K, Duong T, Ferris C (2005). The neural consequences of repeated cocaine exposure revealed by functional MRI in awake rats. Neuropsychopharmacology.

[B137] Ferris CF, Kulkarni P, Sullivan MJJ, Harder JA, Messenger TL, Febo M (2005). Pup suckling is more rewarding than cocaine: Evidence from fMRI and 3D computational analyses. J Neurosci.

[B138] Febo M, Segarra AC, Tenney JR, Sullivan R, Brevard M, Duong TQ, Ferris CF (2004). Imaging cocaine-induced changes in the reward system in conscous rate. J Neurosci Methods.

[B139] Skoubis PD, Hradil VP, Chin CL, Luo Y, Fox GB, McGaraughty S (2006). Mapping brain activity following administration of a nicotinic acetylcholine receptor agonist, ABY-594, using functional magnetic resonance imaging in awake rats. Neuroscience.

[B140] Chin CL, Fox GB, Hradil VP, Osinski MA, McGaraughty SP, Skoubis PD, Cox BF, Luo Y (2006). Pharmacological MRI in awake rats reveals neural activity in area postrema and nucleus tractus solitarius: relevance as a potential biomarker for detecting drug-induced emesis. NeuroImage.

[B141] Ervin G, Schmitz S, Nemeroff C, Prange AJ (1981). Thyrotropin-releasing hormone and amphetamine produce different patterns of behavioral excitation in rats. Eur J Pharmacol.

[B142] Baldino F, Cowan A, Geller EB, Adler MW (1979). Effects of antipsychotic and antianxiety drugs on the morphine abstinence syndrome in rats. J Pharmacol Exp Ther.

[B143] Hashiguchi H, Ye S, Morris M, Alexander N (1997). Single and repeated environmental stress: effect on plasma oxytocin, corticosterone, catecholamines, and behavior. Physiol Behav.

[B144] Brevard ME, Duong TQ, King JA, Ferris CF (2003). Changes in MRI signal intensity during hypercapnic challenge under conscious and anesthetized conditions. Magn Reson Imaging.

[B145] Lahti KM, Ferris CF, Li F, Sotak CH, King JA (1998). Imaging brain activity in conscious animals using functional MRI. J Neurosci Methods.

[B146] Lahti KM, Ferris CF, Li F, Sotak CH, King JA (1999). Comparison of evoked cortical activity in conscious and propofol-anesthetized rats using functional MRI. Magn Reson Med.

[B147] Ludwig R, Bodgdanov G, King J, Allard A, Ferris CF (2004). A dual RF resonator system for high-field functional magnetic resonance imaging of small animals. J Neurosci Methods.

[B148] Sicard K, Shen Q, Brevard ME, Sullivan R, Ferris CF, King JA, Duong TQ (2003). Regional cerebral blood flow and BOLD responses in conscious and anesthetized rats under basal and hypercapnic conditions: implications for functional MRI studies. J Cereb Blood Flow Metab.

[B149] Tenney J, Duong T, King J, Ferris CF (2004). Functional MRI of brain activity in a genetic rat model of absence seizures. Epilepsia.

[B150] Gozzi A, Ceolin L, Schwarz A, Reese T, Bertani S, Crestan V, Bifone A (2007). A multimodality investigation of cerebral hemodynamics and autoregulation in pharmacological MRI. Magn Reson Imaging.

[B151] Qiao M, Rushforth D, Wang R, Shaw R, Tomanek B, Dunn J, Tuor U (2007). Blood-oxygen-level-dependent magnetic resonance signal and cerebral oxygenation responses to brain activation are enhanced by concurrent transient hypertension in rats. J Cereb Blood Flow Metab.

[B152] Wang B, Foniok T, Wamsteeker J, Qiao M, Tomanek B, Vivanco R, Tuor U (2006). Transient blood pressure changes affect the functional magnetic resonance imaging detection of cerebral activation. Neuroimage.

[B153] Kalisch R, Delfinao M, Murer M, Auer D (2005). The phenylephrine blood pressure clamp in pharmacologic magnetic resonance imaging: reduction of systemic confounds and improved detectability of drug-induced BOLD signal changes. Psychopharmacology (Berl).

[B154] Ohata M, Takei H, Fredericks W, Rapoport S (1982). Effects of immobilization stress on cerebral blood flow and cerebrovascular permeability in spontaneously hypertensive rats. J Cereb Blood Flow Metab.

[B155] Hernandez M, Brennan R, Bowman G (1978). Cerebral blood flow autoregulation in the rat. Stroke.

[B156] Hoffman W, Edelman G, Kochs E, Werner C, Segil L, Albrecht R (1991). Cerebral autoregulation in awake versus isoflurane-anesthetized rats. Anesth Analg.

[B157] Sokrab T, Johansson B (1989). Regional cerebral blood flow in acute hypertension induced by adrenaline, noradrenaline and phenylephrine in the conscious rat. Acta Physiol Scand.

[B158] Kelley P, Sharkey J, Philip R, Ritchie IM (1993). Acute cocaine alters cerebrovascular autoregulation in the rat neocortex. Brain Res Bull.

[B159] Febo M, Numan M, Ferris CF (2005). Functional magnetic resonance imaging shows oxytocin activates brain regions associated with mother-pup bonding during sucking. J Neursoci.

[B160] Berwick J, Martin C, Martindale J, Jones M, Johnston D, Zheng Y, Redgrave P, Mayhew J (2002). Hemodynamic response in the unanesthetized rat: intrinsic optical imaging and spectroscopy of the barrel cortex. J Cereb Blood Flow Metab.

[B161] Peeters RR, Tindemans I, De Schutter E, Linden A Van der (2001). Comparing BOLD fMRI signal changes in the awake and anesthetized rat during electrical forepaw stimulation. Magn Reson Imaging.

[B162] Martin C, Martindale J, Berwick J, Mayhew J (2006). Investigating neural – hemodynamic coupling and the hemodynamic response function in the awake rat. NeuroImage.

[B163] Shtoyerman E, Arieli A, Slovin H, Vanzetta I, Grinvald A (2000). Long-term optical imaging and spectroscopy reveal mechanisms underlying the intrinsic signal and stability of cortical maps in V1 of behaving monkeys. J Neurosci.

[B164] Armstrong-James M, George MJ (1988). Influence of anesthesia on spontaneous activity and receptive field size of single units in rat Sm1 neocortex. Exp Neurol.

[B165] Chapin JK, Lin RC (1984). Mapping the body representation in the SI cortex of anesthetized and awake rats. J Comp Neurol.

[B166] Nicolelis MA, Baccala LA, Lin RC, Chapin JK (1995). Sensorimotor encoding by synchronous neural ensemble activity at multiple levels of the somatosensory system. Science.

[B167] Nicolelis M, Lin R, Woodward D, Chapin J (1993). Induction of immediate spatiotemporal changes in thalamic networks by peripheral block of ascending cutaneous information. Nature.

[B168] Krupa DJ, Ghazanfar AA, Nicolelis MA (1999). Immediate thalamic sensory plasticity depends on corticothalamic feedback. Proc Natl Acad Sci USA.

[B169] Fanselow EE, Nicolelis MA (1999). Behavioral modulation of tactile responses in the rat somatosensory system. J Neurosci.

[B170] Bao S, Chan VT, Merzenich MM (2001). Cortical remodelling induced by activity of ventral tegmental dopamine neurons. Nature.

[B171] Rutkowski RG, Weinberger NM (2005). Encoding of learned importance of sound by magnitude of representational area in primary auditory cortex. Proc Natl Acad Sci USA.

[B172] Tolias AS, Sultan F, Augath M, Oeltermann A, Tehovnik EJ, Schiller PH, Logothetis NK (2005). Mapping cortical activity elicited with electrical microstimulation using fMRI in the macaque. Neuron.

[B173] Bard CP (1928). A diencephalic mechanism for the expression of rage with special reference to the sympathetic nervous system. Am J Physiol.

[B174] Ferris CF, Melloni RH, Koppel G, Perry KW, Fuller RW, Delville Y (1997). Vasopressin/serotonin interactions in the anterior hypothalamus control aggressive behavior in golden hamsters. J Neurosci.

[B175] Grimes JM, Melloni RH (2002). Serotonin modulates offensive attck in adolescent anabolic steroid-treated hamsters. Pharmacol Biochem Behav.

[B176] Ricci LA, Rasakham K, Grimes JM, Melloni RH (2006). Serotonin-1A receptor activity and expression modulate adolescent anabolic/androgenic steroid-induced aggression in hamsters. Pharmacol Biochem Behav.

[B177] Ferris CF, Pilapil CG, Hayden-Hixson D, Wiley R, Koh ET (1991). Evidence for two functionally and anatomically distinct populations of magnocellular neurons in the golden hamster. J Neuroendocrinol.

[B178] Delville Y, Mansour KM, Ferris CF (1996). Serotonin blocks vasopressin-facilitated offensive aggression: interactions within the ventrolateral hypothalamus of golden hamsters. Physiol Behav.

[B179] Altemus M, Cizza G, Gold PW (1992). Chronic fluoxetine treatment reduces hypothalamic vasopressin secretion in vitro. Brain Res.

[B180] Hajnal JV, Myers R, Oatridge A, Schwieseo JE, Young IR, Bydder GM (1994). Artifacts due to stimulus correlated motion in functional imaging of the brain. Magn Reson Med.

[B181] Yetkin FZ, Haughton VM, Cox RW, Hyde J, Birn RM, Wong EC, Prost R (1996). Effect of motion outside the field of view on functional MR. AJNR Am J Neuroradiol.

[B182] Birn RM, Bandettini PA, Cox RW, Jesmanowicz A, Shaker R (1998). Magnetic field changes in the human brain due to swallowing or speaking. Magn Reson Med.

[B183] Turner R, Howseman A, Rees G, Josephs O, Frackowiak RSJ (1997). Functional imaging with magnetic resonance. Human Brain Function.

[B184] Arnauld E, Czernichow P, Fumoux F, Vincent JD (1977). The effects of hypotension and hypovolaemia on the liberation of vasopressin during haemorrhage in the unanesthetized monkey. Pfluegers Arch.

[B185] Scremin OU, Paxinos G (1995). Cerebral vascular system. The Rat Nervous System.

[B186] Woods RP, Cherry SR, Mazziotta JC (1992). Rapid automated algorithm for aligning and reslicing PET images. J Comput Assist Tomogr.

[B187] Woods R, Grafton S, Holmes C, Cherry S, Mazziotta J (1998). Automated image registration: I. General methods and intrasubject, intramodality validation. J Comput Assist Tomogr.

[B188] Woods R, Grafton S, Watson J, Sicotte N, Mazziotta J (1998). Automated image registration: II. Intersubject validation of linear and nonlinear models. J Comput Assist Tomogr.

[B189] Cox RW (1996). AFNI: software for analysis and visualization of functional magnetic resonance neuroimages. Comput Biomed Res.

[B190] Friston KJ, Williams SC, Howard R, Frackowiak RS, Turner R (1996). Movement-related effects in fMRI time-series. Magn Reson Med.

[B191] Freire L, Mangin JF (2001). Motion correction algorithms may create spurious brain activations in the absence of subject motion. NeuroImage.

[B192] Johnstone T, Ores Walsh KS, Greischar LL, Alexander AL, Fox AS, Davidson RJ, Oakes TR (2006). Motion correction and the use of motion covariates in multiple-subject fMRI analysis. Hum Brain Mapp.

[B193] Strupp JP (1996). Stimulate:a GUI based fMRI analysis software package. Neuroimage.

[B194] Genovese CR, Lazar NA, Nichols T (2002). Thresholding of statistical maps in functional neuroimaging using the false discovery rate. NeuroImage.

[B195] Hubel DH, Wiesel TN (1959). Receptive fields of single neurones in the cat's striate cortex. J Physiol.

[B196] Rakic P (2002). Evolving concepts of cortical radial and areal specification. Prog Brain Res.

[B197] Boas DA, Jones SR, Devor A, Huppert TJ, Dale AM (2008). A vascular anatomical network model of the spatio-temporal response to brain activation. Neuroimage.

[B198] Harel N, Lee SP, Nagaoka T, Kim DS, Kim SG (2002). Origin of negative blood oxygenation level-dependent fMRI signals. J Cereb Blood Flow Metab.

[B199] Shmuel A, Augath M, Oeltermann A, Logothetis NK (2006). Negative functional MRI response correlates with decreases in neuronal activity in monkey visual area V1. Nat Neurosci.

[B200] Shmuel A, Yacoub E, Pfeuffer J, Moortele PF Van de, Adriany G, Hu X, Ugurbil K (2002). Sustained negative BOLD, blood flow and oxygen consumption response and its coupling to the posiitive response in the human brain. Neuron.

[B201] Devor A, Ulbert I, Dunn AK, Narayanan SN, Jones SR, Anderrmann ML, Boas DA, Dale AM (2005). Coupling of the cortical hemodynamic response to cortical and thalamic neuronal activity. Proc Natl Acad Sci USA.

[B202] Devor A, Tian P, Nishimura N, Teng IC, Hillman EM, Narayanan SN, Ulbert I, Boas DA, Kleinfeld D, Dale AM (2007). Suppressed neuronal activity and concurrent arteriolar vasoconstriction may explain negative blood oxygenation level-dependent signal. J Neurosci.

[B203] de Bruin JP, van Oyen HG, Poll N Van de (1983). Behvioural changes following lesions of the orbital prefrontal cortex in male rats. Behav Brain Res.

[B204] Best M, Williams JM, Coccaro EF (2002). Evidence for a dysfunctional prefrontal circuit in patients with an impulsive aggressive disorder. Proc Natl Acad Sci USA.

[B205] Gammie SC, Negron A, Newman SM, Rhodes JS (2004). Corticotropin-releasing factor inhibits maternal aggression in mice. Behavioral Neuroscience.

[B206] Davis ES, Marler C (2004). c-Fos changes following aggressive encounter in female California mice: a synthesis of behavior, hormone changes and neural activity. Neuroscience.

[B207] Gobrogge KL, Liu Y, Jia X, Wang Z (2007). Anterior hypothalamic neural activation and neurochemical associations with aggression in pair-bonded male prairie voles. The Journal of Comparative Neurology.

[B208] Miczek KA, Brykczynski T, Grossman SP (1974). Differential effects of lesions in the amygdala, periamygdaloid cortex, and stria terminalis on aggressive behaviors in rats. J Comp Physiol Psychol.

[B209] Rosvold HE, Mirsky AF, Pribram KH (1954). Influence of amygdalectomy on social behavior in monkeys. J Comp Physiol Psychol.

[B210] Potegal M, Hebert M, DeCoster M, Meyerhoff J (1996). Brief, high-frequency stimulation of the corticomedial amygdala induces a delayed and prolonged increase of aggressiveness in male Syrian golden hamsters. Behavioral Neuroscience.

[B211] Martinez M, Phillips PJ, Herbert JL (1998). Adaptation in patterns of c-fos expression in the brain associated with exposure to either single or repeated social stress in male rats. European Journal of Neuroscience.

[B212] Veening JG, Coolen LM, de Jong TR, Joosten HW, De Boer SF, Koolhaas JM, al e (2005). Do similar neural systems subserve aggressive and sexual behavior in male rats? Insights from c-fos and pharmacological studies. Eur J Pharmacol.

[B213] Kollack-Walker S, Newman SW (1995). Mating and agonistic behavior produce different patterns of Fos immunolabeling in the male Syrian hamster brain. Neuroscience.

[B214] Gammie SC, Nelson RJ (2001). c-FOS and p-CREB activation and maternal aggression in mice. Brain Research.

[B215] Joppa MA, Meisel RL, Garber MA (1995). c-Fos expression in female hamster brain following sexual and aggressive behaviors. Neuroscience.

[B216] Luiten PGM, Koolhaas JM, De Boer SF, Koopmans SJ (1985). The cortico-medial amygdala in the central nervous system organization of agonistic behavior. Brain Research.

[B217] Halasz J, Liposits Z, Meelis W, Kruk MR, Haller J (2002). Hypothalamic attack area-mediated activation of the forebrain in aggression. Neuroreport.

[B218] King MB, Hoebel BG (1968). Killing elicited by brain stimulation in rats. Communication in Behavioral Biology.

[B219] Bergquist EH (1970). Output pathways of hypothalamic mechanisms for sexual, aggressive and other motivated behaviors in opossum. J Comp Physiol Psychol.

[B220] DeSisto MJ, Huston JP (1971). Aggression and reward from stimulating common sites in the posterior lateral hypothalamus of rats. Communication in Behavioral Biology.

[B221] Panksepp J (1971). Aggression elicited by electrical stimulation of the hypothalamus in albino rats. Physiol Behav.

[B222] Woodworth CH (1971). Attack elicited in rats by electrical stimulation of the lateral hypothalamus. Physiol Behav.

[B223] Bermond B, Mos J, Meelis W, Poel AM van der, Kruk MR (1982). Aggression induced by stimulation of the hypothalamus: Effects of androgens. Pharmacol Biochem Behav.

[B224] Kruk MR, Poel AM Van der, Meelis W, Hermans J, Mostert PG, Mos J, Lohman AHM (1983). Discriminant analysis of the localization of aggression-inducing electrode placements in the hypothlamus of male rats. Brain Research.

[B225] Lammers JHCM, Kruk MR, Meelis W, Poel AM Van der (1988). Hypothalamic substrates for brain stimulation-induced attack, teeth-chattering and social grooming in the rat. Brain Research.

